# Uncovering the diversity of monogeneans (Platyhelminthes) on endemic cypriniform fishes of the Balkan Peninsula: new species of *Dactylogyrus* and comments on their phylogeny and host-parasite associations in a biogeographic context

**DOI:** 10.1051/parasite/2020059

**Published:** 2020-11-24

**Authors:** Eva Řehulková, Michal Benovics, Andrea Šimková

**Affiliations:** 1 Department of Botany and Zoology, Faculty of Science, Masaryk University Kotlářská 2 611 37 Brno Czech Republic

**Keywords:** Monogenea, Dactylogyridae, *Dactylogyrus*, Cyprinidae, Leuciscidae, Balkan Peninsula

## Abstract

Seven new species of *Dactylogyrus* Diesing, 1850 (Dactylogyridae) are described from the gills of seven endemic species of cyprinoids (Cyprinidae, Leuciscidae) inhabiting the Balkan Peninsula: *Dactylogyrus romuli* n. sp. from *Luciobarbus albanicus* (Greece), *Dactylogyrus remi* n. sp. from *Luciobarbus graecus* (Greece), *Dactylogyrus recisus* n. sp. from *Pachychilon macedonicum* (Greece), *Dactylogyrus octopus* n. sp. from *Tropidophoxinellus spartiaticus* (Greece), *Dactylogyrus vukicae* n. sp. from *Delminichthys adspersus* (Bosnia and Herzegovina), *Dactylogyrus leptus* n. sp. from *Chondrostoma knerii* (Bosnia and Herzegovina), and *Dactylogyrus sandai* n. sp. from *Telestes karsticus* (Croatia). To delineate species boundaries, we used an integrative taxonomic approach combining morphological and genetic data. With these tools, we illustrate that some species of monogeneans considered as cryptic might be designated as pseudocryptic (morphologically similar, not easily differentiated) after a posteriori detailed morphological examination, as happened with *D. romuli* n. sp. and *D. remi* n. sp. Thus, for accurate species characterization, it is particularly important to acquire both morphological and molecular data from the same individual specimens, ideally along with illustrations of taxonomically important structures directly taken from hologenophores. Using phylogenetic reconstruction, we investigated the phylogenetic position of newly described *Dactylogyrus* species within *Dactylogyrus* species from Balkan cyprinoids with regard to morphological characteristics, host range, and geographical distribution.

## Abstract (fr)

Sept nouvelles espèces de *Dactylogyrus* Diesing, 1850 (Dactylogyridae) sont décrites à partir des branchies de sept espèces endémiques de cyprinoïdés (Cyprinidae, Leuciscidae) habitant la péninsule balkanique : *Dactylogyrus romuli* n. sp. de *Luciobarbus albanicus* (Grèce), *Dactylogyrus remi* n. sp. de *Luciobarbus graecus* (Grèce), *Dactylogyrus recisus* n. sp. de *Pachychilon macedonicum* (Grèce), *Dactylogyrus octopus* n. sp. de *Tropidophoxinellus spartiaticus* (Grèce), *Dactylogyrus vukicae* n. sp. de *Delminichthys adspersus* (Bosnie-Herzégovine), *Dactylogyrus leptus* n. sp. de *Chondrostoma knerii* (Bosnie-Herzégovine) et *Dactylogyrus sandai* n. sp. de *Telestes karsticus* (Croatie). Pour la délimitation des espèces, nous avons utilisé une approche taxonomique intégrative combinant des données morphologiques et génétiques. Avec ces outils, nous illustrons que certaines espèces de Monogènes considérées comme cryptiques pourraient être désignées comme pseudocryptiques (morphologiquement similaires, pas facilement différenciées) après un examen morphologique détaillé *a posteriori*, comme cela s’est produit avec *D. romuli* n. sp. et *D. remi* n. sp. Ainsi, pour une caractérisation précise des espèces, il est particulièrement important d’acquérir des données morphologiques et moléculaires à partir des mêmes spécimens individuels, idéalement avec des illustrations de structures taxonomiquement importantes directement prises à partir d’hologénophores. En utilisant la reconstruction phylogénétique, nous avons étudié la position phylogénétique des espèces de *Dactylogyrus* nouvellement décrites au sein des espèces de *Dactylogyrus* des cyprinoïdés des Balkans en ce qui concerne les caractéristiques morphologiques, la gamme d’hôtes et la distribution géographique.

## Introduction

The Mediterranean basin of the Balkan Peninsula is recognised as a global biodiversity hotspot [49]. The fauna of this region is characterized by the exceptionally high diversity and endemism of freshwater fishes, especially Cypriniformes [40, 50]. In the last few decades, a number of investigations using molecular data have been devoted to investigate the relationships among Balkan cypriniform taxa and to infer their biogeographical histories [8, 9, 52, 87]. However, only a few studies [3, 4, 77] have focused on investigating the distribution and phylogeny of their host-specific parasites, such as monogeneans, which may reflect the evolutionary history of their associated cypriniform hosts.

Parasites with a high degree of host-specificity are generally expected to show close co-evolutionary relationships with their hosts [39, 55]. As monogeneans have a direct life-cycle and exhibit relatively high host-specificity, they represent powerful model organisms to search for a link between host and parasite diversification in ecological and evolutionary contexts [56]. Among monogeneans, *Dactylogyrus* Diesing, 1850 (Dactylogyridae) is the most speciose genus, with more than 900 nominal species described mostly from the gills of cyprinoids [28]. Morphological characters of the sclerites of the attachment organ (i.e., haptor) as well as of the copulatory organs (i.e., male copulatory organ and vagina) are commonly used for monogenean species identification [58]. In recent years, however, a number of previously unrecognized (morphologically cryptic) parasite species have been revealed using molecular markers [57]. Within monogeneans, a molecular approach has been applied especially to delimitate and describe “morphologically simple” species of *Gyrodactylus* von Nordmann, 1832 (Gyrodactylidae) (e.g. [42, 61, 92]), whose taxonomy depends primarily on the shape of the haptoral structures, especially the marginal hooks [27, 45, 70]. Although *Dactylogyrus* species have many accessible morphological characters (i.e. those of the haptor and the reproductive system) compared to *Gyrodactylus* spp., cryptic species within this genus have also been detected. Recently, Rahmouni et al. [59] reported two cryptic *Dactylogyrus* species (*D. benhoussai* Rahmouni, Řehulková & Šimková, 2017 and *D. varius* Rahmouni, Řehulková & Šimková, 2017) in association with two allopatrically distributed species of *Luciobarbus* (*L. yahyaouii* Doadrio, Casal-López & Perea and *L. maghrebensis* Doadrio, Perea & Yahyaoui, respectively) in Morocco. Benovics et al. [4] revealed potential complexes of cryptic species within three *Dactylogyrus* species (*D. rutili* Gläser, 1965, *D. dyki* Ergens & Lucký, 1959 and *D. ergensi* Molnar, 1964) parasitizing Balkan cyprinids.

In terms of host specificity, the majority of *Dactylogyrus* species are highly host-specific. However, the host specificity of *Dactylogyrus* species may range from strict specialism (i.e. species restricted to a single host species) and intermediate specialism (i.e. species restricted to a single host genus) to intermediate generalism (i.e. species parasitizing phylogenetically related non-congeneric hosts) and true generalism (i.e. species parasitizing phylogenetically unrelated hosts) [76]. On the basis of the list of *Dactylogyrus* species identified by Benovics et al. [4] on endemic cyprinoids from the Balkans, we can calculate that each species parasitizes a mean number of 1.2 host genera; 94% of the species are reported from a single genus. As many *Dactylogyrus* species are restricted to a single host species, members of this genus may provide valuable models to obtain novel insights into host ecology and evolution [72].

As a result of two independent colonization events [8, 26, 53, 80, 91], as well as the influence of the reforming of the Dessaretes Lake system in the past [1, 2, 69, 85, 88], the Balkan Peninsula harbors remarkable species diversity among freshwater fishes belonging to the suborder Cyprinoidei. Currently, 17 highly diversified genera of cyprinoids are recognized in the Balkans. Nevertheless, some genera exhibit high morphological similarity and/or close phylogenetic relationships, i.e. *Telestes* and *Squalius* or *Barbus* and *Luciobarbus* [40, 52, 68]. *Barbus* and *Squalius* are the genera with the highest species richness in the Balkans and are the only genera distributed across all southern European Peninsulas [53, 67, 68]. *Chondrostoma* species are distributed in the northern Mediterranean drainages across Europe, western Asia, and the Middle East [21], while those of *Telestes* inhabit only the Balkan and Apennine Peninsulas [9, 40]. In contrast, the species distributions of *Delminichthys*, *Pachychilon*, *Pelasgus* and *Tropidophoxinellus* are limited to several river streams of lake systems in the Balkans [40]. Although only two species of *Luciobarbus* (former *Barbus* [86, 87]) are native of the Balkans, they are associated with two different evolutionary lineages. While *L. albanicus* (Steindachner) is phylogenetically closer to the Middle Eastern and North African species of *Luciobarbus*, *L. graecus* (Steindachner) is closely related to the Iberian lineage of *Luciobarbus* spp. and to *L. lydianus* (Boulenger) from Turkey [89].

To date, only a limited number of studies have been focused on investigating the diversity and/or phylogenetic relationships of *Dactylogyrus* spp. parasitizing the Balkan cypriniform fishes [3, 4, 18, 19, 71, 81–84]. Recently, Benovics et al. [4] revealed eight potentially new *Dactylogyrus* species on two species of cyprinids (*Luciobarbus albanicus* and *L. graecus*) and six species of leuciscids (*Chondrostoma knerii* Heckel, *Delminichthys adspersus* (Heckel), *Pachychilon macedonicum* (Steindachner), *Squalius tenellus* Heckel, *Tropidophoxinellus spartiaticus* (Schmidt-Ries), and *Telestes karsticus* Marčić & Mrakovčić). In the present paper, we provide descriptions of seven of these eight *Dactylogyrus* spp. using a combined morphological and molecular approach.

## Materials and methods

### Fish sampling

**Figure 1:**
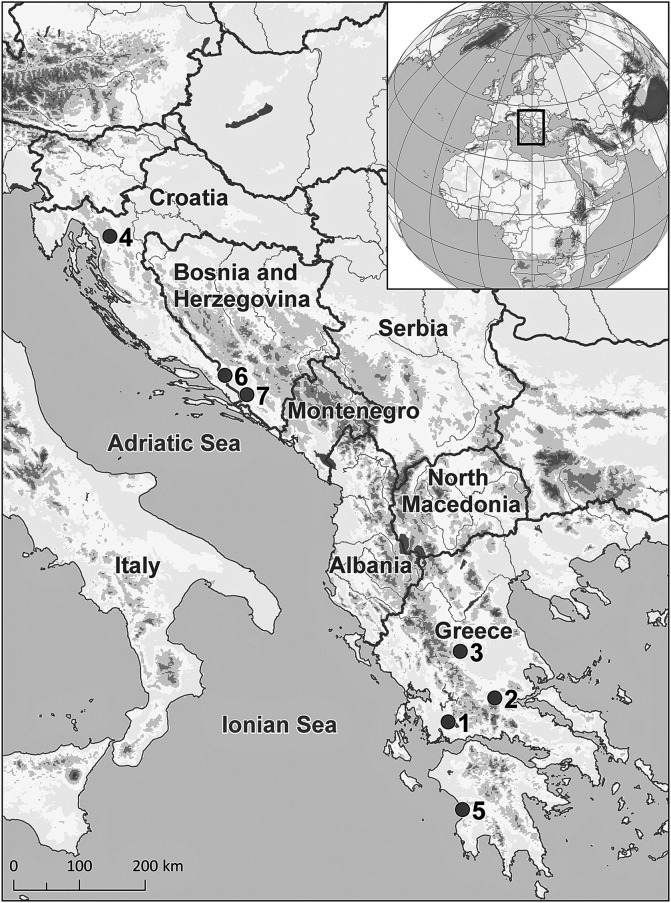
Map showing the sampling localities (numbers) for each host species of the Balkan Peninsula near the eastern coast of the Adriatic and Ionian Sea. (1) Trichonis Lake, Panetolio; (2) Spercheios River, Ypati; (3) Pineios River, Rongia – Valamandrio; (4) Sušik River, Drežnica; (5) Neda River, Giannitsochori; (6) Nezdravica River, Tihaljina; (7) Rečina River, Hutovo Blato Natural Park.

Fifty-nine individuals from seven species representing six genera of cypriniforms (Cyprinidae and Leuciscidae) were collected by means of gill nets or electro-fishing from seven localities in Bosnia and Herzegovina, Croatia, and Greece, during the period 2014–2017 (Fig. 1). Sampling was carried out under field approvals MPK BR: 07/1/625-342/15/ (Bosnia and Herzegovina), URBROJ: 517 -07 -l-l-l-16-4 (Croatia) and HCMR: 220965/2583/22-8-2011 (Greece). Fish hosts were identified *in situ* using the key provided by Kottelat and Freyhof [40], and the identification was subsequently confirmed using sequences of the cytochrome *b* mitochondrial gene recorded by Radek Šanda (National Museum, Prague, Czech Republic) and Jasna Vukić (Charles University, Prague, Czech Republic). Scientific names and the classification of fishes are those provided in Fricke et al. [25]. Live fishes were kept in aerated holding tanks until they were processed for parasitological examination; fishes were sacrificed by severing the spinal cord.

### Parasite sampling

**Figure 2:**
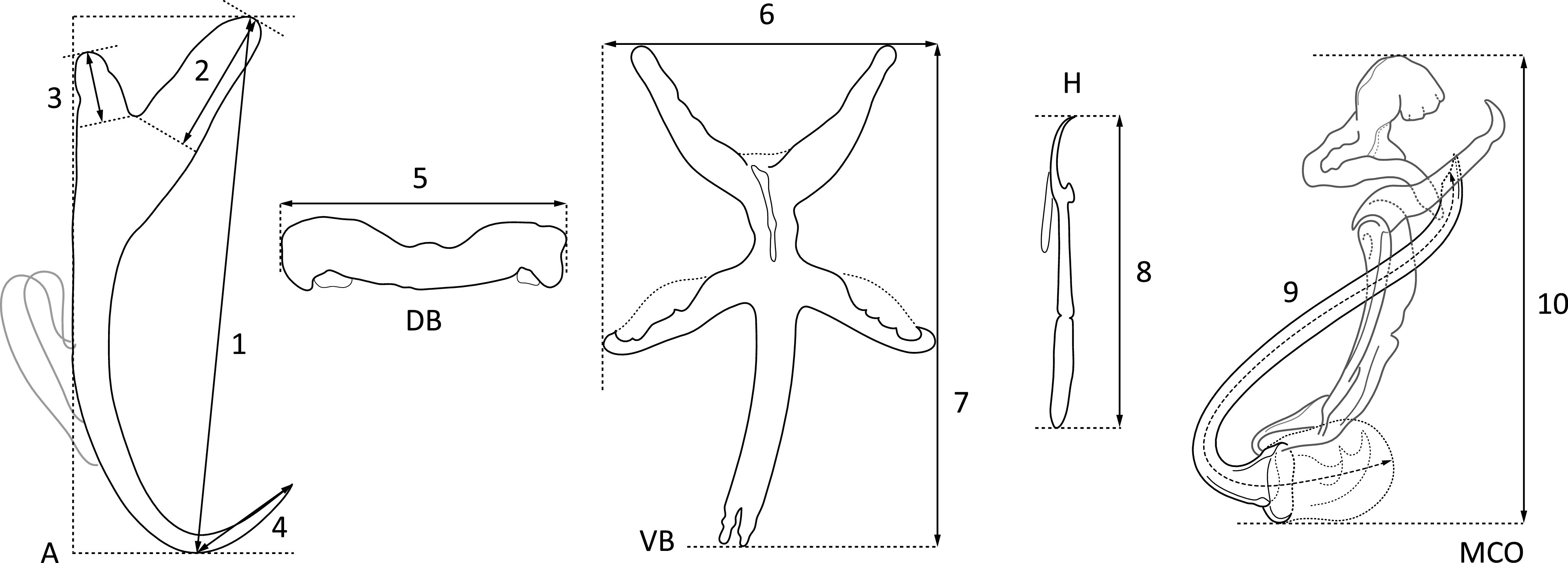
Scheme of measurements for sclerotized structures of *Dactylogyrus* spp. A, anchor (1 – total length, 2 – inner root length, 3 – outer root length, 4 – point length); DB, dorsal bar (5 – width); VB, ventral bar (6 – width, 7 – length); H, hook (8 – length); MCO, male copulatory organ (9 – tube curved length; 10 – total straight length).

Gills of freshly killed fishes were extracted, placed in a Petri dish containing water, and examined using a SZX7 stereomicroscope (Olympus, Tokyo, Japan). Monogeneans were removed from the gills using fine needles and prepared as in Řehulková [62]. Specimens used for morphological examination were completely flattened under coverslip pressure in order to best expose their sclerotized structures (haptoral and reproductive sclerites), and fixed with a mixture of glycerine and ammonium picrate (= GAP; Malmberg [44]). Specimens used for DNA analysis were bisected using fine needles. Subsequently, one half of the body (either the posterior part with haptoral sclerites or anterior part containing the male copulatory organ) was fixed in 96% ethanol for later DNA extraction, and the remaining half was mounted on a slide, fixed with GAP for species identification, and kept as a hologenophore (i.e. a voucher specimen from which a molecular sample is directly derived (*sensu* Pleijel et al. [54]). The mounted specimens (or their parts) were studied using a BX61 microscope (Olympus, Tokyo, Japan) equipped with phase-contrast optics. Drawings of the sclerotized structures were made with the aid of a drawing attachment and edited with a graphic tablet (Wacom Intuos5 Touch) compatible with Adobe Illustrator and Adobe Photoshop (Adobe Systems Inc., San Jose, CA, USA). Measurements were taken using Stream Motion 1.9.2 image analysis software (Olympus). The list of measurements for the sclerites is shown in Figure 2. All measurements (in micrometres) are provided as the mean followed by the range and the number (*n*) of specimens measured in parentheses. The numbering of hook pairs (in Roman numerals I-VII) is that recommended by Mizelle [48]. The male copulatory organ is henceforth abbreviated to MCO. After morphometric analysis, the specimens (or their parts) fixed with GAP were dehydrated and re-mounted in Canada balsam as permanent mounts, according to Ergens [22]. Type and voucher specimens collected during the present study were deposited at the Institute of Parasitology of the Czech Academy of Sciences (IPCAS), České Budějovice, Czech Republic and the Muséum National d’Histoire Naturelle (MNHN), Paris, France as indicated in the respective species accounts. To comply with the regulations set out in article 8.5 of the amended 2012 version of the International Code of Zoological Nomenclature [35], details of the new species have been submitted to ZooBank. Epidemiological characteristics such as parasite prevalence (percentage of infected hosts), abundance (mean number of parasites per host taking into account both infected and uninfected individuals), and intensity of infection (minimum and maximum number of parasites per infected host) were calculated for each *Dactylogyrus* species according to Bush et al. [10].

### DNA extraction, amplification, and sequencing

Bisected *Dactylogyrus* individuals preserved in ethanol were dried using a vacuum centrifuge. DNA was extracted using the standard protocol (DNeasy Blood & Tissue Kit, Qiagen, Hilden, Germany). Partial 18S ribosomal DNA, the entire ITS1 region, and partial 5.8S ribosomal DNA were amplified using the primers S1 and IR8 following Šimková et al. [74]. DNA amplification was performed following the protocol and conditions described in Benovics et al. [4]. Partial 28S ribosomal DNA was amplified using the primers C1 and D2 following Hassouna et al. [34]. DNA amplification was performed using the same conditions as described in Šimková et al. [75]. The purification of PCR and subsequent sequencing follow Benovics et al. [4]. Sequencing was performed on an ABI 3130 Genetic Analyzer (Applied Biosystems). Gene sequences of the new *Dactylogyrus* species were generated as part of larger phylogenetic studies [4, 77] and have been deposited in GenBank previously (their accession numbers are in Table 1).

**Table 1:** List of *Dactylogyrus* species, their cyprinoid host species, country of collection, and GenBank accession numbers for sequences used for the phylogenetic reconstruction.

*Dactylogyrus* species	Host species	Country	28S rDNA	18S rDNA+ITS1
*D. anchoratus* (Dujardin, 1845)	*Carassius gibelio*	Czech Republic	KY863555	KY859795
*D. balkanicus* Dupont & Lambert, 1986	*Barbus prespensis*	Greece	KY201107	KY201093
*D. borealis* Nybelin, 1937	*Phoxinus* sp.	Bosnia-Herzegovina	KY629372	KY629343
*D. caucasicus* Mikailov & Shaova, 1937	*Alburnoides devoli*	Albania	MG792954	MG792840
*D. crivellius* Dupont & Lambert, 1986	*Barbus prespensis*	Greece	KY201108	KY201094
*D. dirigerus* Gussev, 1966	*Chondrostoma vardarensis*	Greece	MG792992	MG792877
*D. dyki* Ergens & Lucký, 1959	*Barbus cyclolepis*	Greece	MG792971	MG792856
*D. ergensi* Molnár, 1964	*Chondrostoma vardarensis*	Greece	MG792993	MG792878
*D. folkmanovae* Ergens, 1956	*Squalius cephalus*	Bosnia-Herzegovina	MG793028	MG792911
*D. formosus* Kulwiec, 1927	*Carassius gibelio*	Croatia	MG792984	MG792869
*D. leptus* n. sp.	*Chondrostoma knerii*	Bosnia-Herzegovina	MG792986	MG792871
*D. martinovici* Ergens, 1970	*Pachychilon pictum*	Greece	MG793001	MG792885
*D. octopus* n. sp.	*Tropidophoxinellus spartiaticus*	Greece	MG793065	MG792950
*D. omenti* Benovics et al. 2017	*Aulopyge huegelii*	Bosnia-Herzegovina	KY201105	KY201091
*D. petenyi* Kašťák, 1957	*Barbus cyclolepis*	Greece	MG792972	MG792857
*D. petkovici* Ergens, 1970	*Pachychilon pictum*	Greece	MG793003	MG792887
*D. recisus* n. sp.	*Pachychilon macedonicum*	Greece	MG792998	MG792882
*D. remi* n. sp.	*Luciobarbus graecus*	Greece	KY201115	KY201101
*D. romuli* n. sp.	*Luciobarbus albanicus*	Greece	KY201114	KY201100
*D. rosickyi* Ergens, 1970	*Pachychilon pictum*	Greece	MG793004	MG792888
*D. rutili* Gläser, 1965	*Rutilus lacustris*	Greece	MG793016	MG792900
*D. rysavyi* Ergens, 1970	*Alburnoides thessalicus*	Greece	MG792965	MG792851
*D. sandai* n. sp.	*Telestes karsticus*	Croatia	MG793057	MG792942
*D. sekulovici* Ergens, 1970	*Pachychilon pictum*	Greece	MG793005	MG792889
*D. suecicus* Nybelin, 1937	*Rutilus lacustris*	Greece	MG793017	MG792901
*D. tissensis* Zakhvatkin, 1951	*Alburnoides thessalicus*	Greece	MG792966	MG792852
*D. vranoviensis* Ergens, 1956	*Squalius squalus*	Bosnia-Herzegovina	MG793048	MG792931
*D. vukicae* n. sp.	*Delminichthys adspersus*	Bosnia-Herzegovina	MG792995	MG792881

### Phylogenetic reconstruction

Sequences of partial 18S rDNA, combined with the ITS1 region, and partial 28S rDNA were used for phylogenetic reconstruction. DNA sequences were aligned using the fast Fourier transform algorithm implemented in MAFFT [38]. Final alignments were trimmed manually to unify the length of all sequences. The final nucleotide sequence alignment contained sequences of 27 species (19 previously described, 7 new and one unnamed) of *Dactylogyrus* from the Balkans obtained from GenBank, and two *Dactylogyrus* species from *Carassius gibelio* (Bloch) (namely *D. anchoratus* (Dujardin, 1845) and *D. formosus* Kulwiec, 1927), which were used as outgroup for rooting the phylogenetic trees (accession numbers in Table 1). JModelTest 2.1.10 [14, 30] was employed to infer the optimal evolutionary model for each genetic segment (18S, ITS1 and 28S respectively), using the Bayesian information criterion (BIC) as the penalization algorithm. Maximum likelihood (ML) and Bayesian inference (BI) analyses were conducted in RaxML v.8.2.11 [78, 79] and MrBayes 3.2.6 [66], respectively. Internal node support for the ML tree was assessed by running 1000 bootstrap pseudoreplicates. Two parallel runs, each with four Markov chains, were executed for BI analysis and run for 1,000,000 generations. Trees were sampled every 100 generations and the first 30% of the resulting trees were discarded as initial burn-in. Convergence was indicated by an average standard deviation of split frequencies per parallel run of <0.01, and subsequently checked using Tracer v. 1.7.1 [60]. Posterior probabilities were calculated as the frequency of samples recovering particular clades. To uncover the genetic divergence between morphologically similar species, uncorrected pair-wise genetic distances (*p*-distances) were calculated for each genetic segment in MEGA7 [41]. The same species as those used in phylogenetic reconstruction analyses (excluding species used as outgroup) were used for the computation of genetic distances. The final alignments contained sequences of 27 *Dactylogyrus* species (see Supplementary Tables 1–3).

## Results

**Figure 3:**
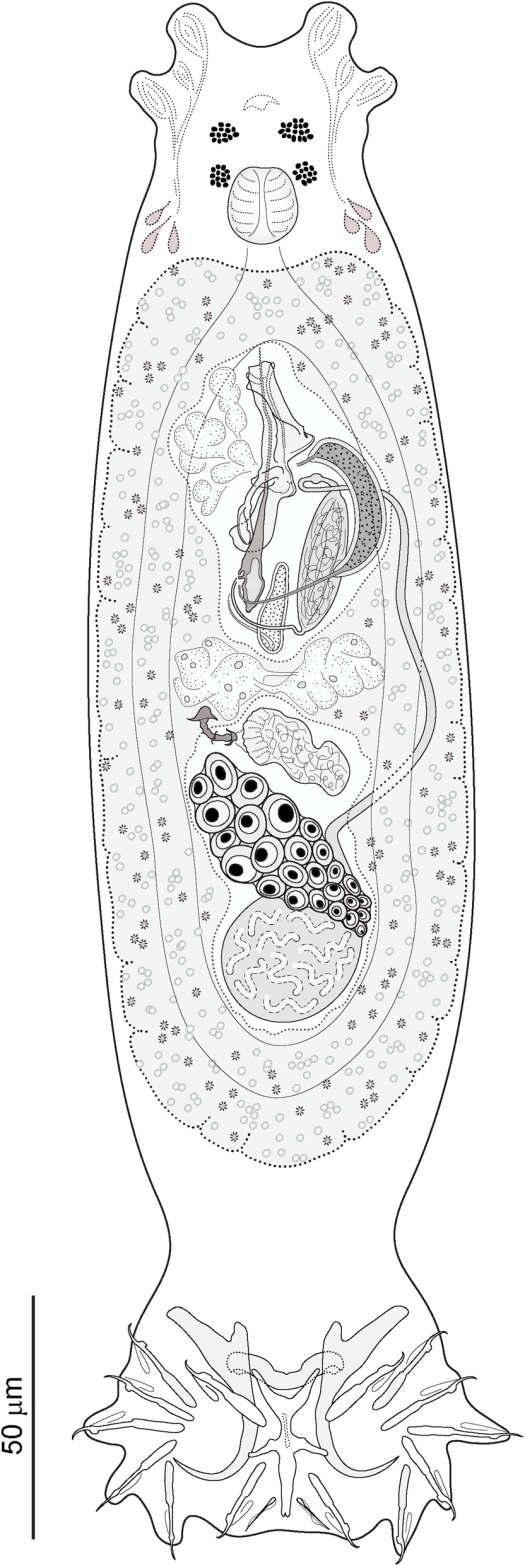
*Dactylogyrus remi* n. sp. Full-body composite drawing, ventral view.

Seven cyprinoid fish species from the Balkan Peninsula were examined for monogeneans: *Chondrostoma knerii* (*n* = 5; 165–200 mm in total length); *Delminichthys adspersus* (*n* = 10; 58–66 mm in total length); *Luciobarbus albanicus* (*n* = 4; 70–173 mm in total length); *L. graecus* (*n* = 10; 86–156 mm in total length); *Pachychilon macedonicum* (*n* = 8; 61–88 mm in total length); *Telestes karsticus* (*n* = 10; 66–85 mm in total length), and *Tropidophoxinellus spartiaticus* (*n* = 5; 84–116 mm in total length). Each species was infected with one species of *Dactylogyrus*. Generic assignment of species was based on the combined presence of the following characters (see Fig. 3 for illustration of *D. remi* n. sp.): (1) thin and smooth tegument; (2) body divided into a cephalic area, trunk, peduncle and haptor; (3) cephalic margin with two bilateral lobes; (4) two pairs of eyes comprised of ovate granules; accessory eye granules may be present in parts or throughout body; (5) mouth subterminal, ventral; (6) pharynx spherical to ovate; (7) gut bifurcate; ceca without diverticula, confluent posteriorly; (8) gonads tandem or overlapping (testis postovarian); (9) vas deferens looping left intestinal cecum; (10) seminal vesicle a dilated portion of vas deferens; (11) two prostatic reservoirs present; (12) male copulatory organ (MCO) composed of copulatory tube and accessory piece; (13) ovary ventral to testis; (14) seminal receptacle a proximal dilation of vagina; (15) vagina dextroventral; (16) vitellarium coextensive with gut; (17) haptor possessing one pair of anchors (pointed to the dorsal side of the haptor) with bases connected by a dorsal bar, seven pairs of hooks with normal distribution [48], and one pair of needle-like structures lying near hooks of pair V; ventral bar present or absent.

**Figure 4:**
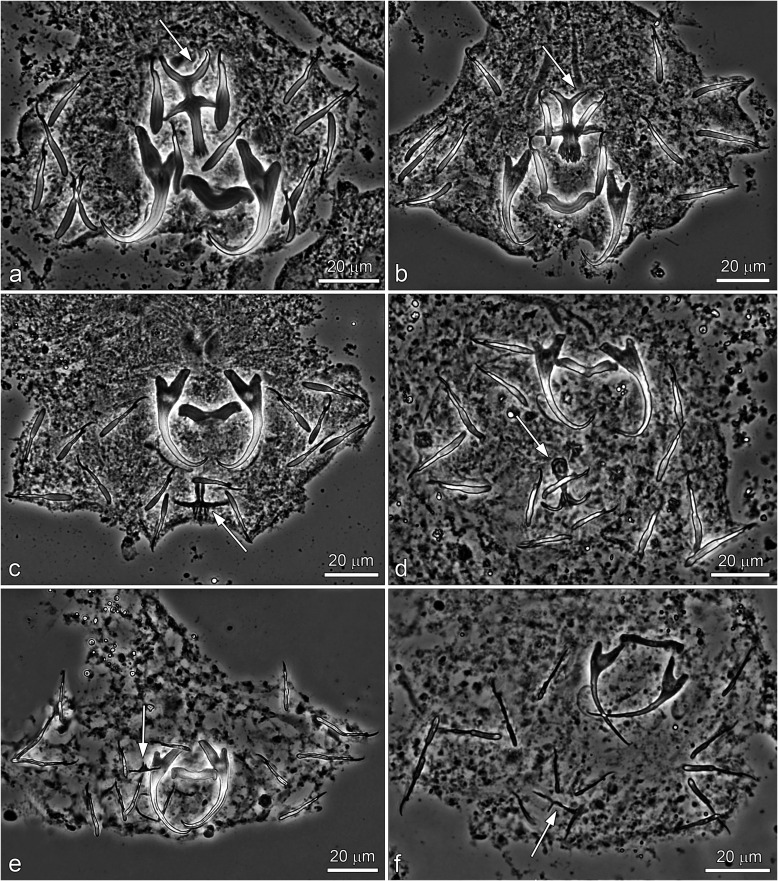
Phase-contrast micrographs of the sclerotized haptoral structures of *Dactylogyrus* spp. Arrows indicate the shape/type of the ventral bars. (a) *D. romuli* n. sp. (five radial type with long terminally frayed posterior arm), (b) *D. recisus* n. sp. (five radial type with posterior arm shorter than anterior one and with deeply frayed termination), (c) *D. sandai* n. sp. (cross-shaped type with posterior arm longitudinally split into several rays), (d) *D. octopus* n. sp. (inverted T-shaped type with pyriform anterior arm), (e) *D. vukicae* n. sp. (inverted T-shaped type with anterior arm reduced into several finger-like rays), (f) *D. leptus* n. sp. (poorly sclerotized, inverted T-shaped type with pointed arms of the same size).

New species (see below) were described mainly on the basis of (i) phase contrast microscopic examination of the sclerotized structures (i.e. those of the haptor and distal parts of the female and male reproductive systems) and (ii) assessment of sequence divergence accompanied by phylogenetic analysis in order to evaluate relationships between new and selected known *Dactylogyrus* spp. The seven new *Dactylogyrus* species identified can be classified into six different morphological types based on the haptoral bars (see Fig. 4).

### *Dactylogyrus romuli* n. sp. (Figs. 4a, 5, 6a, 13)

urn:lsid:zoobank.org:act:9EDA14B8-AD33-4D6A-90B9-3ABF58BC2BB4

**Figure 5:**
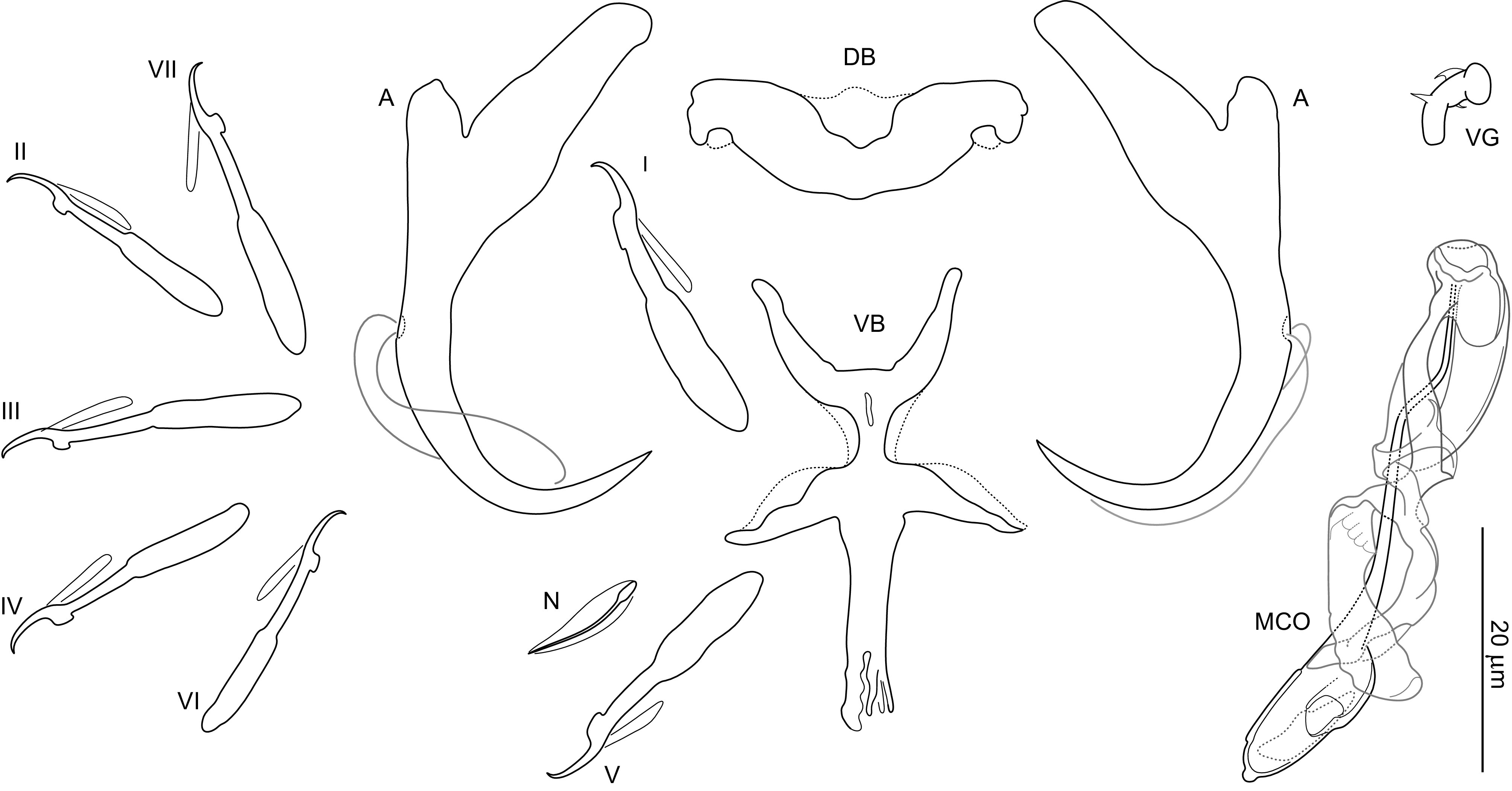
Sclerotized structures of *Dactylogyrus romuli* n. sp. ex *Luciobarbus albanicus*. A, anchor; DB, dorsal bar; VB, ventral bar; N, needle; I–VII, hooks; VG, vagina; MCO, male copulatory organ.

**Figure 6:**
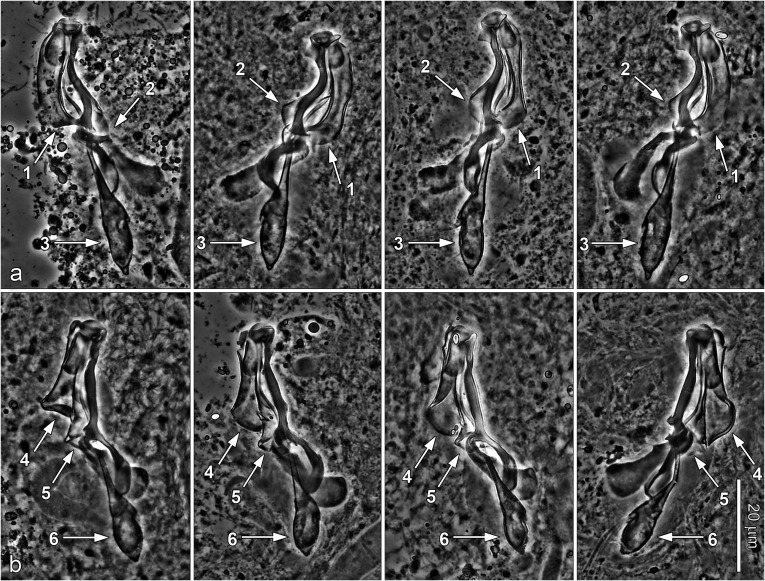
Phase-contrast micrographs of the MCOs in *Dactylogyrus romuli* n. sp. (a) and *D. remi* n. sp. (b). Characters of interest are indicated by arrows: (1) distal fold without expanded proximal portion, (2) medial fold on the opposite side to the distal fold, (3) base markedly elongated, (4) distal fold with expanded proximal portion (cornet shaped), (5) medial fold on the same side as the distal fold, (6) base slightly elongated.

*Type host and locality: Luciobarbus albanicus* (Steindachner, 1870); Trichonis Lake (Acheloos Basin), Panetolio (38°35′20.19″ N 21°28′02.68″ E), Greece (June 2014).

*Site on host:* Gill lamellae.

*Prevalence, mean abundance and intensity range of infection*: 75% (3 fish infected/4 fish examined); 102.5; 17–279 monogeneans per infected host.

*Type and voucher material:* Holotype, 5 paratypes and hologenophore voucher (IPCAS M-711); paratype (MNHN HEL1337) and hologenophore voucher (MNHN HEL1338).

*Representative DNA sequences*: A nucleotide sequence of partial 28S rDNA (795 bp; KY201114) and nucleotide sequences representing a fragment (1024 bp; KY201100) including partial 18S rDNA (488 bp), the ITS1 region (530 bp) and 5.8S (6 bp). No intraspecific variability was found (6 specimens were analyzed).

*Etymology:* This species is named for Romulus, the legendary founder of Rome and the twin brother of Remus, and refers to the resemblance with the below-named species.

*Description* (based on 10 specimens): With characters of the genus as defined by Gussev [32]. Body 368 (279–533; *n* = 5) long; greatest width 75 (79–112; *n* = 5) near midlength. Pharynx spherical to ovate; greatest width 26 (22–31; *n* = 4). Haptor 60 (50–79; *n* = 4) long, 98 (71–154; *n* = 4) wide. Anchors with elongate terminally flattened inner root, moderately developed outer root, medially slightly constricted bent shaft, elongate point extending past level of tip of inner root; total length 42 (38–49; *n* = 10); inner root length 17 (15–19; *n* = 10); outer root length 4 (4–5; *n* = 10); point length 9 (8–11; *n* = 10). Anchor filaments well developed. Dorsal bar 27 (25–31; *n* = 10) wide, saddle-shaped. Ventral bar 26 (23–27; *n* = 10) wide, 37 (33–42; *n* = 10) long, cross-shaped; anterior arm widely bifurcated into two long branches, with medial delicate aperture; posterior arm with frayed termination. One pair of needles located near hooks of pair V. Hooks with delicate point, depressed thumb, shank comprised of 2 subunits (proximal subunit expanded); hook pair I with markedly flattened thumb, robust; hook lengths: pair I 26 (25–28; *n* = 5), pair II 22 (21–23; *n* = 5), pair III 25 (24–26; *n* = 5), pair IV 24 (23–25; *n* = 5), pair V 25 (24–27; *n* = 5), pair VI 22 (22–24; *n* = 5), pair VII 27 (26–28; *n* = 5). FH loop extending to near union of shank subunits. MCO composed of basally articulated copulatory tube and accessory piece; total length 52 (46–60; *n* = 10). Copulatory tube 52 (48–61; *n* = 10) long; base elongated oval, with small aperture protruding from its distal part; shaft with subterminal double-bend. Accessory piece complex; proximal half bifurcated into two unequal branches curved toward each other like claws (grooved branch articulated to base of the tube); distal half membranous, folded. Vagina mushroom-shaped, with delicate short filaments.

*Remarks:* Morphologically, *Dactylogyrus romuli* n. sp. belongs to the group of congeners having a ventral bar derived from the cross-shaped type, where the anterior arm is more or less bifurcated and the posterior arm (often elongated) is split or terminally frayed (= the five radial type). These include *Dactylogyrus* species hitherto recorded on cyprinoids (mostly species of *Barbus* and *Luciobarbus*) from rivers draining into the Persian Gulf and the Mediterranean, Black, Caspian and Aral seas (in Morocco and a region stretching from southern France to Central Asia; [58]). Four of these species (i.e. *D. jamansajensis* Osmanov, 1958, *D. deziensis* Gussev, Jalali & Molnar, 1993, *D. deziensioides* Gussev, Jalali & Molnar, 1993, and *D. persis* Bychowsky, 1949) possess MCO of the “kulwieci” type (see [58]), which shows some similarities with the MCO of *D. romuli* n. sp., specifically: (1) the copulatory tube is composed of a relatively robust base and slightly waved shaft; (2) the proximal half of the accessory piece is bifurcated into two branches (one is attached to the base of the tube). Nevertheless, the MCO of *D. romuli* n. sp. is easily differentiated from those of *D. deziensis* and *D. deziensioides* (both from *Luciobarbus kersin* (Heckel), Iran; [33]) by its copulatory tube having a markedly longer and thinner shaft. It clearly differs from the MCOs of *D. jamansajensis* (from *Luciobarbus capito* (Güldenstädt) and *Barbus cyri* De Filippi, Central Asia, Kazakhstan, East Transcaucasus; [51]) and *D. persis* (from *Carasobarbus luteus* (Heckel), Iran; [11]) by possessing an accessory piece with robust claw-shaped proximal branches of similar size (the branches are of unequal size in *D. jamansajensis*, and notably smaller and not claw-shaped in *D. persis*). *Dactylogyrus romuli* n. sp. closely resembles *D. remi* n. sp.; however, there are some differences in MCO morphology (see Remarks for the latter species and Fig. 6 for comparison). In addition, these two species clearly differ at the molecular level, with dissimilarities identified at the ITS1 (5.1%) and 28S rDNA (2.3%) sites.

### *Dactylogyrus remi* n. sp. (Figs. 3, 6b, 7, 13)

urn:lsid:zoobank.org:act:E1E5044A-7A66-4947-929C-2963130C8015

**Figure 7:**
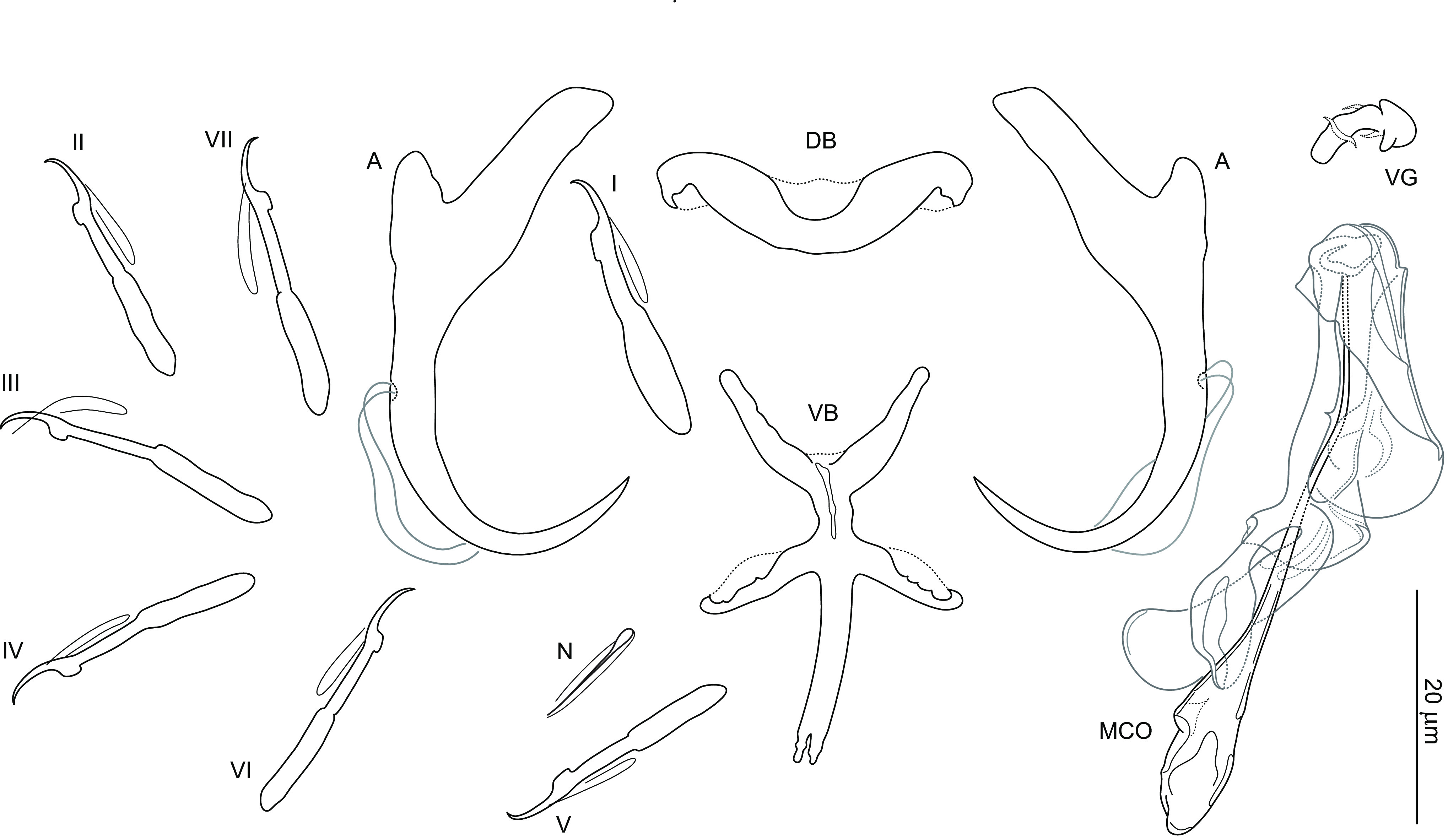
Sclerotized structures of *Dactylogyrus remi* n. sp. ex *Luciobarbus graecus*. A, anchor; DB, dorsal bar; VB, ventral bar; N, needle; I–VII, hooks; VG, vagina; MCO, male copulatory organ.

*Type host and locality*: *Luciobarbus graecus* (Steindachner, 1895); Sperchios River (Sperchios Basin), Ypati (38°54′14.33″ N 22°17′30.22″ E), Greece (June 2014, September 2017).

*Site on host*: Gill lamellae.

*Prevalence, mean abundance and intensity range of infection*: 90% (9 fish infected/10 fish examined); 123.4; 12–687 monogeneans per infected host.

*Type and voucher material*: Holotype, paratype and hologenophore voucher (IPCAS M-712); 3 paratypes (MNHN HEL1339) and hologenophore voucher (MNHN HEL1340).

*Representative DNA sequences*: A nucleotide sequence of partial 28S rDNA (793 bp; KY201115) and nucleotide sequences representing a fragment (982 bp; KY201101) including partial 18S rDNA (487 bp), the ITS1 region (489 bp) and 5.8S (6 bp). No intraspecific variability was found (3 specimens were analyzed).

*Etymology*: This species is named for Remus, the legendary founder of Rome and the twin brother of Romulus, and refers to the resemblance with the above-named species.

*Description* (based on 11 specimens): With characters of the genus as defined by Gussev [32]. Body 298 (220–356; *n* = 9) long; greatest width 68 (40–105; *n* = 9) near midlength. Pharynx spherical to ovate; greatest width 15 (10–17; *n* = 9). Haptor 50 (41–57; *n* = 9) long, 83 (67–96; *n* = 8) wide. Anchors with elongate terminally flattened inner root, moderately developed outer root, medially slightly constricted bent shaft and elongate point extending past level of tip of inner root; total length 40 (37–44; *n* = 11); inner root length 15 (14–16; *n* = 11); outer root length 5 (4–6; *n* = 11); point length 11 (10–12; *n* = 11). Anchor filaments well developed. Dorsal bar 27 (25–29; *n* = 11) wide, saddle-shaped. Ventral bar 24 (21–26; *n* = 11) wide, 34 (32–35; *n* = 11) long, cross-shaped; anterior arm widely bifurcated into two long branches, with medial delicate aperture; posterior arm terminally bifurcated or frayed. One pair of needles located near hooks of pair V. Hooks with delicate point, depressed thumb, shank comprised of 2 subunits (proximal subunit expanded); hook pair I with flattened thumb, robust; hook lengths: pair I 24 (23–25; *n* = 5), pair II 22 (21–23; *n* = 5), pair III 25 (24–26; *n* = 5), pair IV 24 (23–25; *n* = 5), pair V 22 (21–23; *n* = 5), pair VI 23 (22–23; *n* = 5), pair VII 24 (24–25; *n* = 5). FH loop extending to near union of shank subunits. MCO composed of basally articulated copulatory tube and accessory piece; total length 55 (50–63; *n* = 11). Copulatory tube 55 (49–62; *n* = 11) long; base elongated oval, with small aperture protruding from its distal part; shaft with subterminal bend. Accessory piece complex; proximal half bifurcated into 2 branches curved toward each other like claws (grooved branch articulated to the base of the tube); distal half membranous, folded, main fold cornet shaped. Vagina mushroom-shaped, with delicate short filaments.

*Remarks*: *Dactylogyrus remi* n. sp. may be confused with *D. romuli* n. sp. by having nearly identical haptoral structures and very similar copulatory sclerites. In the absence of comparative material, differentiation of the two species may be difficult. However, the comparative morphology of the MCO is the best means of separating the specimens and together with molecular data provide sufficient evidence that the two species are distinct. *Dactylogyrus remi* n. sp. can be distinguished from *D. romuli* n. sp. by its MCO possessing a more robust accessory piece with a cornet-shaped distal fold (the distal fold without the angularly expanded proximal portion in *D. romuli* n. sp.). In addition, the base of the copulatory tube appears to be more elongated in *D. romuli* n. sp. than in *D. remi* n. sp. (see Fig. 6 for comparison).

### *Dactylogyrus recisus* n. sp. (Figs. 4b, 8, 13)

urn:lsid:zoobank.org:act:D33C5461-D540-4389-9BB9-4F1D876C4007

**Figure 8:**
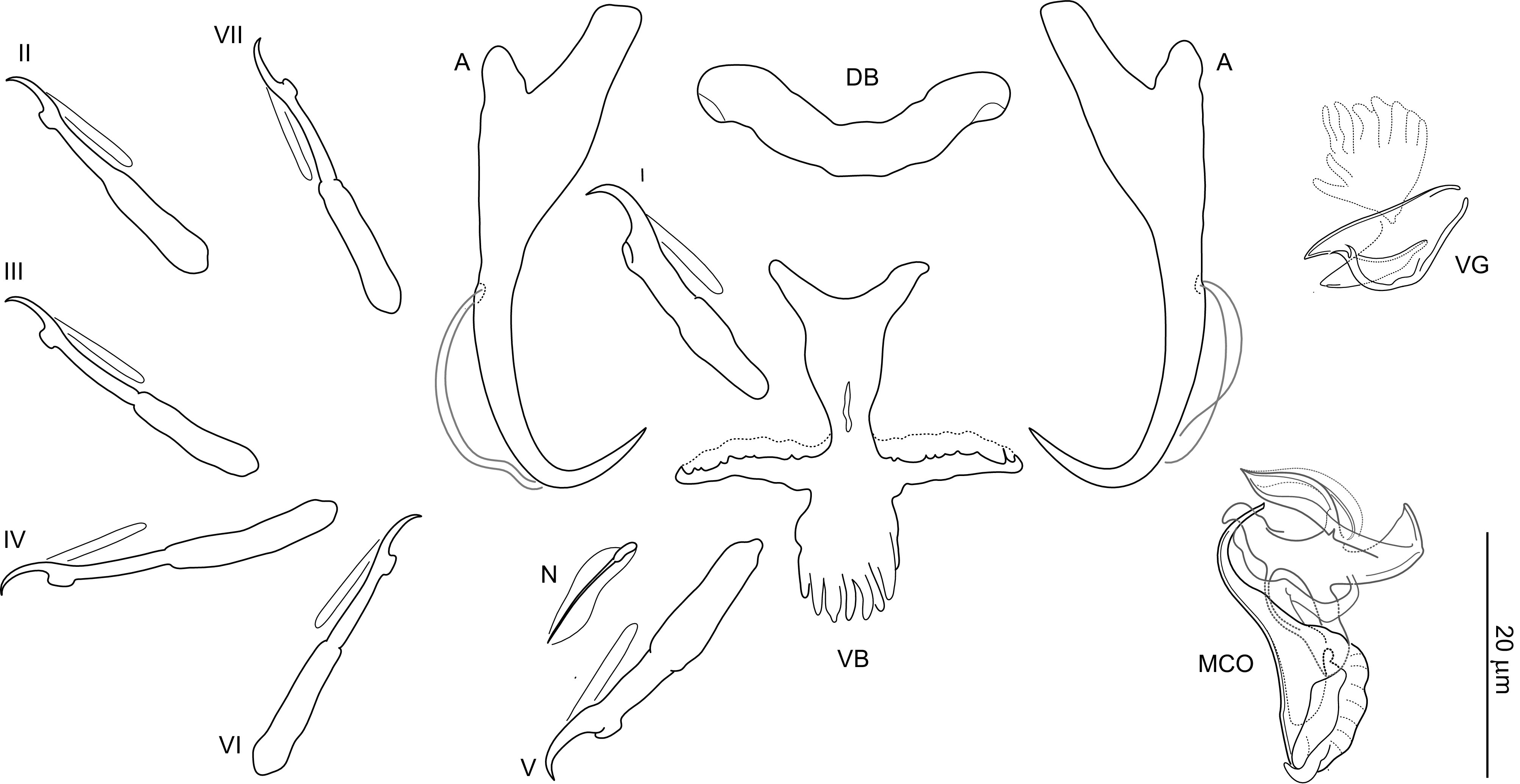
Sclerotized structures of *Dactylogyrus recisus* n. sp. ex *Pachychilon macedonicum*. A, anchor; DB, dorsal bar; VB, ventral bar; N, needle; I–VII, hooks; VG, vagina; MCO, male copulatory organ.

*Type host and locality*: *Pachychilon macedonicum* (Steindachner, 1892); Pinios River (Pinios Basin), Rongia – Valamandrio (39°33′07.85″ N 21°42′08.02″ E), Greece (June 2014).

*Site on host*: Gill lamellae.

*Prevalence, mean abundance and intensity range of infection*: 100% (8 fish examined and infected); 10.9; 3–29 monogeneans per infected host.

*Type and voucher material*: Holotype, paratype and hologenophore voucher (IPCAS M-713); paratype (MNHN HEL1341) and hologenophore voucher (MNHN HEL1342).

*Representative DNA sequences*: A nucleotide sequence of partial 28S rDNA (791 bp; MG792998) and nucleotide sequences representing a fragment (991 bp; MG792882) including partial 18S rDNA (488 bp), the ITS1 region (496 bp) and 5.8S (6 bp). No intraspecific variability was found (9 specimens were analyzed).

*Etymology*: The specific name is from Latin (*recisus* = short, brief), referring to the length of the copulatory tube.

*Description* (based on 9 specimens): With characters of the genus as defined by Gussev [32]. Body 371 (201–554; *n* = 7) long; greatest width 58 (34–87; *n* = 7) near midlength. Pharynx spherical to ovate; greatest width 17 (11–27; *n* = 8). Haptor 67 (48–102; *n* = 5) long, 97 (67–130; *n* = 5) wide. Anchors with moderately developed roots (inner root terminally flattened), straight to slightly bent shaft and point extending just past level of tip of inner root; total length 39 (32–43; *n* = 9); inner root length 10 (9–11; *n* = 9); outer root length 4 (3–4; *n* = 9); point length 8 (7–8; *n* = 9). Anchor filaments well developed. Dorsal bar 24 (21–26; *n* = 9) wide, saddle-shaped. Ventral bar 26 (25–28; *n* = 9) wide, 28 (25–30; *n* = 9) long, cross-shaped; anterior arm widely bifurcated into two short branches, with delicate submedial aperture; posterior arm relatively wide, shorter than the anterior one, with markedly frayed termination. One pair of needles located near hooks of pair V. Hooks with delicate point, depressed thumb, shank comprised of 2 subunits (proximal subunit expanded); hook pairs I, V robust; hook lengths: pair I 21 (19–22; *n* = 5), pair II 22 (19–23; *n* = 5), pair III 24 (21–25; *n* = 5), pair IV 27 (24–29; *n* = 5), pair V 24 (21–26; *n* = 5), pair VI 24 (21–26; *n* = 5), pair VII 23 (21–26; *n* = 5). FH loop extending to near union of shank subunits. MCO composed of basally articulated copulatory tube and accessory piece; total length 21 (21–26; *n* = 9). Copulatory tube 39 (37–40; *n* = 9) long; base massive in comparison to the shaft, oblique cone-shaped, with slightly frilled margins; shaft about same length as base, arcing, markedly tapering terminally. Accessory piece with dove-shaped main part. Vagina scale-like, slightly sclerotized.

*Remarks*: *Dactylogyrus recisus* n. sp. resembles *Dactylogyrus martinovici* Ergens, 1970 and *Dactylogyrus petkovici* Ergens, 1970, both gill parasites of *Pachychilon pictum* (Heckel & Kner) from Lake Skadar [23], in having a cross-shaped ventral bar with an anterior arm widely bifurcated into two short branches and relatively short (i.e. shorter than anterior one) posterior arm with a frayed termination. In addition, all the three species possess anchors with terminally flattened inner roots, slightly bent shafts, and relatively short points. *Dactylogyrus recisus* n. sp. clearly differs from *D. martinovici* and *D. petkovici* by having a copulatory tube with a comparatively shorter shaft and a scale-like vagina (the vagina is coiled in *D. petkovici* and diminutive, discoid in *D. martinovici*).

### *Dactylogyrus sandai* n. sp. (Figs. 4c, 9, 13)

urn:lsid:zoobank.org:act:BF67E989-8FF9-4895-9C44-ECC226E153B1

**Figure 9:**
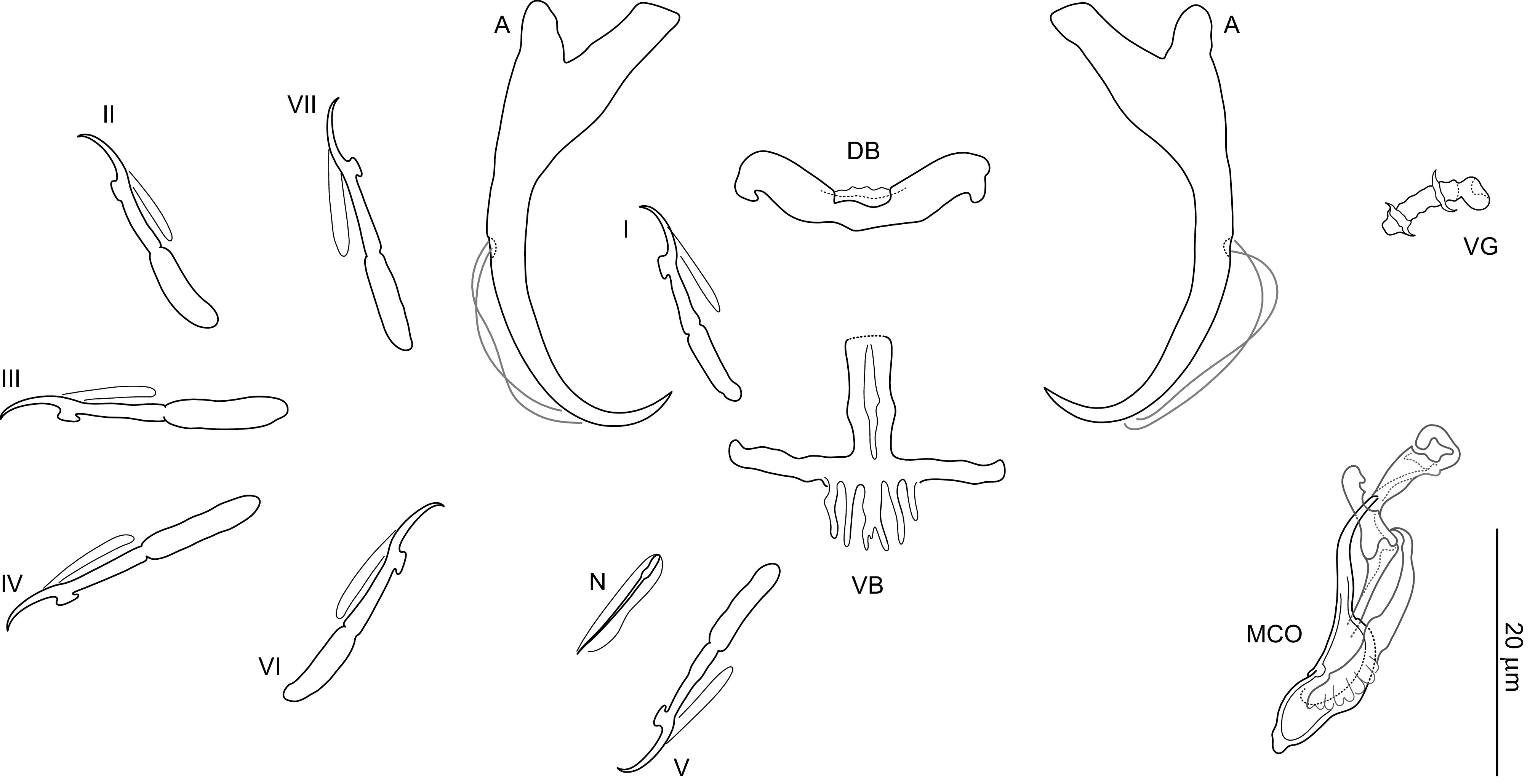
Sclerotized structures of *Dactylogyrus sandai* n. sp. ex *Telestes karsticus*. A, anchor; DB, dorsal bar; VB, ventral bar; N, needle; I–VII, hooks; VG, vagina; MCO, male copulatory organ.

*Type host and locality*: *Telestes karsticus* Marčić & Mrakovčić, 2011; Sušik River (Drežnica Basin), Drežnica (45°08′44.13″ N 15°04′41.56″ E), Croatia (September 2016).

*Site on host*: Gill lamellae.

*Prevalence, mean abundance and intensity range of infection*: 100% (10 fish examined and infected); 12.6; 1–41 monogeneans per infected host.

*Type and voucher material*: Holotype, 4 paratypes, 2 hologenophore vouchers (IPCAS M-714); paratype (MNHN HEL1343) and hologenophore voucher (MNHN HEL1344).

*Representative DNA sequences*: A nucleotide sequence of partial 28S rDNA (790 bp; MG793057) and nucleotide sequences representing a fragment (981 bp; MG792942) including partial 18S rDNA (487 bp), the ITS1 region (494 bp) and 5.8S (6 bp). No intraspecific variability was found (6 specimens were analyzed).

*Etymology*: This species is named after Dr. Radek Šanda, National Museum, Prague, Czech Republic, in recognition of his extensive research on the systematics and phylogeny of Cypriniformes.

*Description* (based on 10 specimens): With characters of the genus as defined by Gussev [32]. Body 349 (229–410; *n* = 6) long; greatest width 62 (52–71; *n* = 6) near midlength. Pharynx spherical to ovate; greatest width 18 (13–22; *n* = 5). Haptor 51 (49–54; *n* = 5) long, 87 (74–100; *n* = 5) wide. Anchors with roots almost equal in size (inner root terminally flattened), slightly bent shaft, and point extending past level of tip of inner root; total length 36 (34–38; *n* = 10); inner root length 10 (8–11; *n* = 10); outer root length 5 (4–5; *n* = 10); point length 6 (5–6; *n* = 10). Anchor filaments well developed. Dorsal bar 22 (19–23; *n* = 10) wide, saddle-shaped. Ventral bar 24 (19–27; *n* = 10) wide, 16 (13–17; *n* = 10) long, cross-shaped; anterior arm with medial longitudinal aperture; posterior arm markedly shorter than anterior one, longitudinally split into several rays (usually about five). One pair of needles located near hooks of pair V. Hooks with delicate point, depressed thumb, shank comprised of 2 subunits (proximal subunit expanded); hook lengths: pair I 18 (18–20; *n* = 5), pair II 19 (19–20; *n* = 5), pair III 24 (23–25; *n* = 5), pair IV 23 (23–25; *n* = 5), pair V 21 (20–22; *n* = 5), pair VI 20 (20–21; *n* = 5), pair VII 22 (21–22; *n* = 5). FH loop extending to near union of shank subunits. MCO composed of basally articulated copulatory tube and accessory piece; total length 29 (28–30; *n* = 10). Copulatory tube 30 (29–31; *n* = 10) long; base massive in comparison with shaft, foot-like, medially slightly constricted, with small apical tubercle; shaft about same length as base, curved, proximally slightly enlarged in diameter, distally tapered. Accessory piece comprising two proximal branches (one with finger-frilled flange appearing to line one side of tube opening) articulated to T-shaped distal part guiding termination of copulatory tube. Vagina a short tube crossed by two bars.

*Remarks*: On the basis of the morphology of the ventral bar, *D. sandai* n. sp. resembles a number of congeners possessing a cross-shaped ventral bar with short (longitudinally deeply frayed) posterior arm and distally (anteriorly) slightly broadening anterior arm (= “rutili” type in Gussev [32]). It most resembles *Dactylogyrus distinguendus* Nybelin, 1937 and *Dactylogyrus rutili* (see [58]). However, unlike in the above two species, the posterior arm of the ventral bar in *D. sandai* n. sp is wider than the anterior arm and split longitudinally into rays. The MCO of *D. sandai* n. sp. most resembles those of the “nanus” type [58] in *Dactylogyrus nanus* Dogiel & Bychowsky, 1934, *Dactylogyrus nanoides* Gussev, 1966, and *Dactylogyrus suecicus* Nybelin, 1937, but clearly differs from them by having a copulatory tube with medially slightly enlarged (*vs.* evenly tapering) shaft and an accessory piece with a T-shaped distal part (*vs.* a leaf sheath-like distal part).

### *Dactylogyrus octopus* n. sp. (Figs. 4d, 10, 13)

urn:lsid:zoobank.org:act:8BC4060B-5802-486B-8C5C-84AB37665DA0

**Figure 10:**
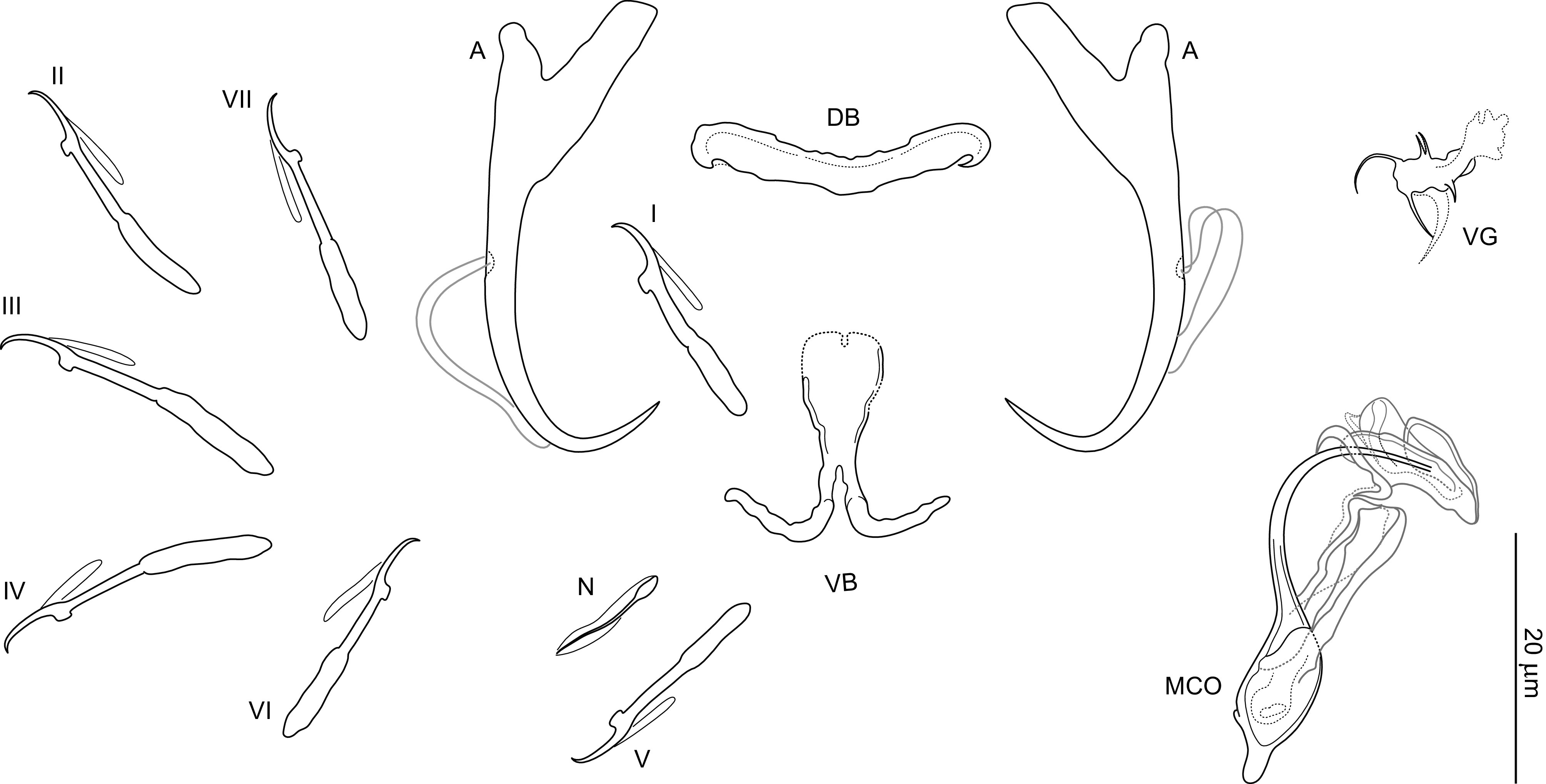
Sclerotized structures of *Dactylogyrus octopus* n. sp. ex *Tropidophoxinellus spartiaticus*. A, anchor; DB, dorsal bar; VB, ventral bar; N, needle; I–VII, hooks; VG, vagina; MCO, male copulatory organ.

*Type host and locality*: *Tropidophoxinellus spartiaticus* (Schmidt-Ries, 1943); Neda River (Neda Basin), Gianitsochori (37°23′04.34″ N 21°41′24.15″ E), Greece (June 2014).

*Site on host*: Gill lamellae.

*Prevalence, mean abundance and intensity range of infection*: 100% (5 fish examined and infected); 13.4; 1–24 monogeneans per infected host.

*Type and voucher material*: Holotype, 4 paratypes and hologenophore voucher (IPCAS M-715); paratype (MNHN HEL1345) and hologenophore voucher (MNHN HEL1346).

*Representative DNA sequences*: A nucleotide sequence of partial 28S rDNA (790 bp; MG793065) and nucleotide sequences representing a fragment (991 bp; MG792950) including partial 18S rDNA (487 bp), the ITS1 region (495 bp) and 5.8S (6 bp). No intraspecific variability was found (5 specimens were analyzed).

*Etymology*: The specific name, a noun, refers to the shape of the ventral bar resembling an octopus.

*Description* (based on 10 specimens): With characters of the genus as defined by Gussev [32]. Body 348 (273–440; *n* = 6) long; greatest width 62 (51–70; *n* = 6) near midlength. Pharynx spherical to ovate; greatest width 16 (12–19; *n* = 6). Haptor 53 (40–59; *n* = 3) long, 78 (68–83; *n* = 3) wide. Anchors with moderately developed roots (inner root terminally flattened), straight to slightly bent shaft and point extending past level of tip of inner root; total length 36 (33–37; *n* = 10); inner root length 9 (7–9; *n* = 10); outer root length 4; point length 8 (7–9; *n* = 10). Anchor filaments well developed. Dorsal bar a slightly curved rod, 24 (21–25; *n* = 10) wide. Ventral bar 19 (17–20; *n* = 10) wide, 18 long (16–19; *n* = 10), three-armed (converse T-shaped); anterior arm pyriform, not bifurcated; transverse arms bent (directed) anterolaterally. One pair of needles located near hooks of pair V. Hooks with delicate point, depressed thumb, shank comprised of 2 subunits (proximal subunit expanded); hook lengths: pair I 20 (20–22; *n* = 5), pair II 21 (20–23; *n* = 5); pair III 25 (24–27; *n* = 5); pair IV 26 (24–29; *n* = 5); pair V 20 (19–20; *n* = 5); pair VI 22 (21–24; *n* = 5); pair VII 22 (21–24; *n* = 5). FH loop about 0.6 times the distal shank length. MCO composed of basally articulated copulatory tube and accessory piece; total length 32 (28–33; *n* = 10). Copulatory tube 41 (37–44; *n* = 10) long; base diamond shaped, with conspicuous apical heel-like projection; shaft arcing, tapering terminally. Accessory piece comprising two filamentous proximal branches, foliaceous distal sheath enclosing distal end of shaft of copulatory tube. Vagina variably spined.

*Remarks*: *Dactylogyrus octopus* n. sp. can be assigned to the group of *Dactylogyrus* spp. with an inverted T-shaped ventral bar. However, the pyriform anterior arm and two transverse arms bent anterolaterally distinguish this new species from all other known members of the genus. On the basis of the morphology of the MCO, *D. octopus* n. sp. most resembles *Dactylogyrus* spp. possessing MCO of the “nanus” type [58], which is characterized by a copulatory tube with a foot-like base (usually with a heel-like apical projection) and relatively short curved shaft, and an accessory piece differentiated into two parts (the proximal part is attached to the base and the distal part is widened, guiding the terminal part of the tube). In *D. octopus* n. sp., the MCO differs from that of the “nanus” type by the following combination of characters: (1) the base of the tube is diamond-shaped rather than foot-like; (2) the basal heel-like projection is large (*vs.* missing or small) and situated in the direction of the shaft’s axis (*vs.* situated more or less laterally to the shaft’s axis).

### *Dactylogyrus vukicae* n. sp. (Figs. 4e, 11, 13)

urn:lsid:zoobank.org:act:F1D46FAA-E0A1-46F9-97C9-0D49DB808F53

**Figure 11:**
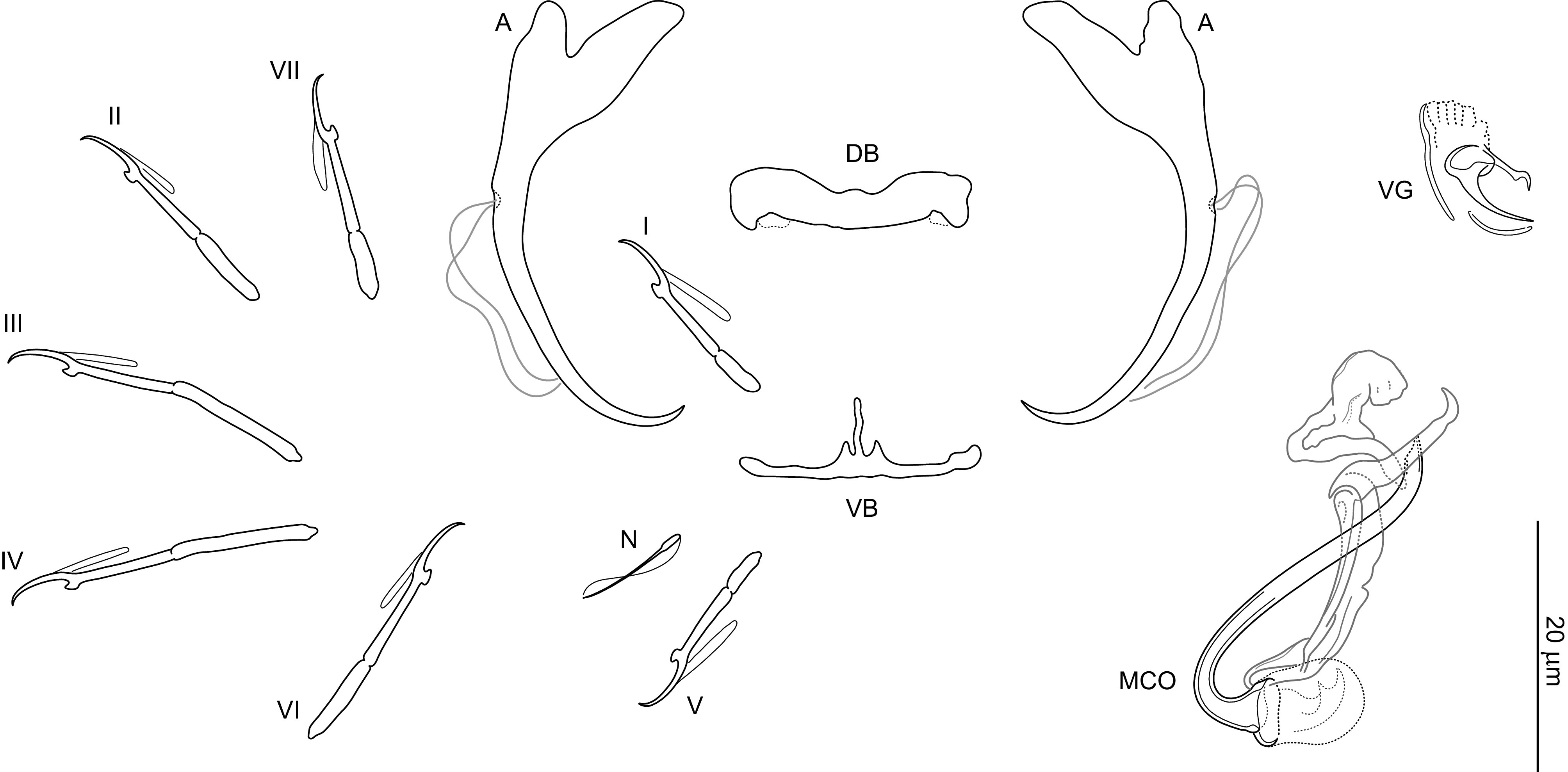
Sclerotized structures of *Dactylogyrus vukicae* n. sp. ex *Delminichthys adspersus*. A, anchor; DB, dorsal bar; VB, ventral bar; N, needle; I–VII, hooks; VG, vagina; MCO, male copulatory organ.

*Type host and locality*: *Delminichthys adspersus* (Heckel, 1843); Nezdravica River (Neretva Basin), Tihaljina (43°19′00.05″ N 17°23′20.01″ E), Bosnia and Herzegovina (July 2015).

*Site on host*: Gill lamellae.

*Prevalence, mean abundance and intensity range of infection*: 100% (10 fish examined and infected); 4; 1–15 monogeneans per infected host.

*Type and voucher material*: Holotype, paratype and hologenophore voucher (IPCAS M-716); paratype (MNHN HEL1347) and hologenophore voucher (MNHN HEL1348).

*Representative DNA sequences*: A nucleotide sequence of partial 28S rDNA (790 bp; MG792995) and nucleotide sequences representing a fragment (980 bp; MG792881) including partial 18S rDNA (487 bp), the ITS1 region (487 bp) and 5.8S (6 bp). No intraspecific variability was found (6 specimens were analyzed).

*Etymology*: This species is named after Dr. Jasna Vukić, Charles University, Prague, Czech Republic, in recognition of his extensive research on the systematics and phylogeny of Cypriniformes.

*Description* (based on 8 specimens): With characters of the genus as defined by Gussev [32]. Body 302 (270–310; *n* = 4) long; greatest width 62 (55–65; *n* = 4) near midlength. Pharynx spherical to ovate; greatest width 20 (18–22; *n* = 4). Haptor 47 (41–52; *n* = 4) long, 71 (67–75; *n* = 4) wide. Anchors with moderately developed roots (inner root terminally flattened), elongate bent shaft (usually medially slightly waved) and short point extending past level of tip of inner root; total length 35 (34–36; *n* = 8); inner root length 10 (*n* = 8); outer root length 4 (3–4; *n* = 8); point length 4 (3–4; *n* = 8). Anchor filaments well developed. Dorsal bar rod-shaped, with medial anterior depression, 20 (19–21; *n* = 8) wide. Ventral bar 20 (19–21; *n* = 8) wide, 5 (4–7; *n* = 5) long, three-armed; anterior arm longitudinally split into two or three variable finger-like rays. One pair of needles located near hooks of pair V. Hooks with delicate point, erect thumb, shank comprised of 2 subunits (proximal subunit expanded); hook lengths: pair I 17 (16–18; *n* = 3); pair II 21 (20–22; *n* = 3); pair III 28 (27–19; *n* = 3); pair IV 28 (28–29; *n* = 3); pair V 18 (17–18; *n* = 3); pair VI 23 (22–24; *n* = 3); pair VII 20 (20–21; *n* = 3). FH loop about 0.5 times the distal shank length. MCO composed of basally articulated copulatory tube and primary accessory piece; total length 33 (32–39; *n* = 8). Copulatory tube 40 (38–43; *n* = 8) long; base bulbous, thin-walled; shaft pipe-shaped. Two accessory pieces; primary accessory piece comprising proximal branch articulated to sickle shaped distal part; secondary accessory piece non-articulated to copulatory tube, appearing as a saddle like structure. Vagina with poorly sclerotized entry.

*Remarks*: The ventral bar of *D. vukicae* n. sp. slightly resembles that of *Dactylogyrus* spp. parasitizing leuciscids of Phoxininae (*D. borealis* Nybelin, 1936, *D. phoxini* Malewitzkaja, 1949, *D. ersinensis* Spassky & Roytman, 1960, *D. malewitzkajae* Gussev, 1955, *D. gvosdevi* Gussev, 1955, *D. amurensis* Akhmerov, 1952, and *D. szerskii* Gussev, 1955) [58]. All of these species possess a delicate inverted T-shaped ventral bar, usually with poorly developed anterior arm. However, unlike in *Dactylogyrus* spp. from *Phoxinus* spp., the anterior arm in *D. vukicae* n. sp. is split longitudinally into two or three rays and the posterior part in the middle of the ventral bar is straight, without a shallow groove. The MCO of the new species resembles that of *Dactylogyrus erhardovae* Ergens, 1970 and *Dactylogyrus crucifer* Wagener, 1857, both parasitizing species of *Rutilus* [58], in that all three species have a J-shaped copulatory tube with a thin-walled base and an accessory piece comprising a proximal branch articulated to a sickle-shaped distal part*. Dactylogyrus vukicae* n. sp. clearly differs from both above species by possessing two accessory pieces, one of which is not articulated to the copulatory tube.

### *Dactylogyrus leptus* n. sp. (Figs. 4f, 12, 13)

urn:lsid:zoobank.org:act:0C30810B-DDAF-4736-A589-32BFA7676385

**Figure 12:**
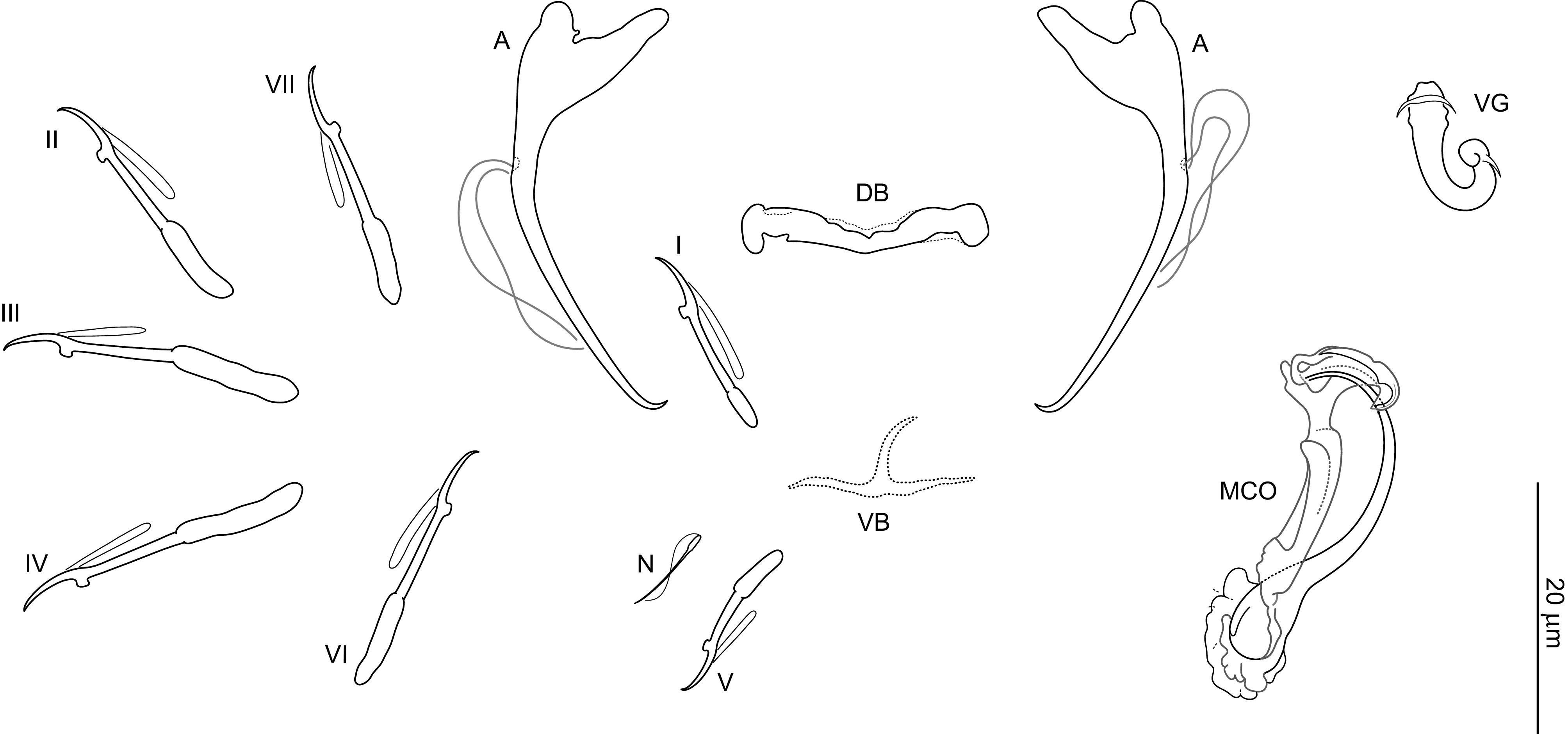
Sclerotized structures of *Dactylogyrus leptus* n. sp. ex *Chondrostoma knerii*. A, anchor; DB, dorsal bar; VB, ventral bar; N, needle; I–VII, hooks; VG, vagina; MCO, male copulatory organ.

**Figure 13:**
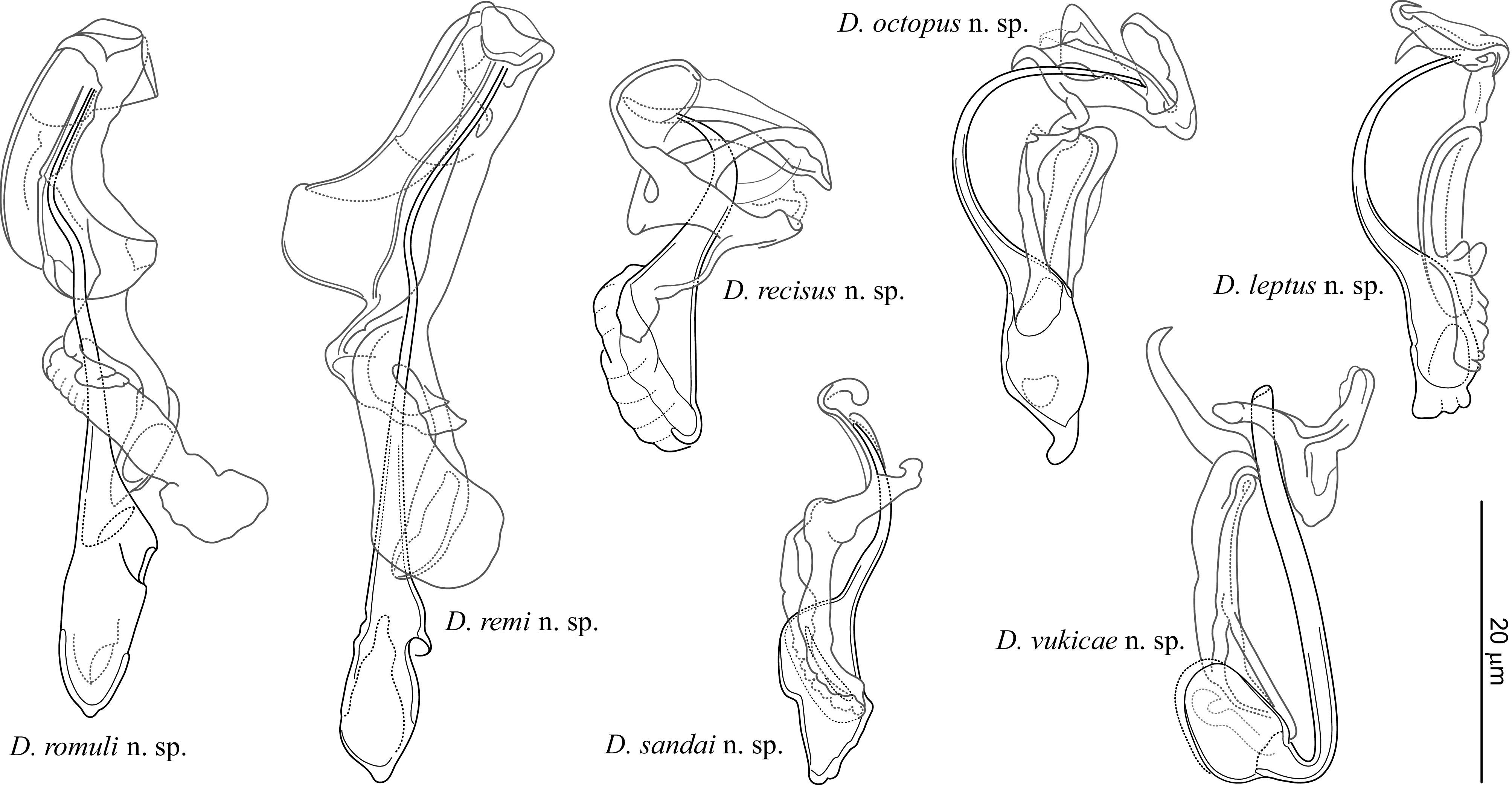
*Dactylogyrus* spp. (MCOs of hologenophores) parasitizing cyprinoids in the Balkan Peninsula.

*Type host and locality*: *Chondrostoma knerii* Heckel, 1843; Rečina River (Neretva Basin), near Jelim Lake, Hutovo Blato National Park (43°03′39.72″ N 17°48′29.30″ E), Bosnia and Herzegovina (October 2016).

*Site on host*: Gill lamellae.

*Prevalence, mean abundance and intensity range of infection*: 80% (4 fish infected/5 fish examined); 2.6; 2–6 monogeneans per infected host.

*Type and voucher material*: Holotype, paratype and 2 hologenophore vouchers (IPCAS M-717); paratype (MNHN HEL1349) and hologenophore voucher (MNHN HEL1350).

*Representative DNA sequences*: A nucleotide sequence of partial 28S rDNA (791 bp; MG792986) and nucleotide sequences representing a fragment (979 bp; MG792871) including partial 18S rDNA (487 bp), the ITS1 region (486 bp) and 5.8S (6 bp). No intraspecific variability was found (9 specimens were analyzed).

*Etymology*: The specific name is from Greek (*leptos* = thin, slender) and refers to the shaft of the anchors.

*Description* (based on 10 specimens): With characters of the genus as defined by Gussev [32]. Body 315 (240–395; *n* = 3) long; greatest width 61 (55–72; *n* = 3) near midlength. Pharynx spherical to ovate; greatest width 17 (12–25; *n* = 3). Haptor 48 (41–56; *n* = 3) long, 74 (68–83; *n* = 3) wide. Anchors with moderately developed roots (sometimes rising further apart), markedly elongate bent shaft and rudimentary point extending past level of tip of inner root; total length 33 (33–34; *n* = 10); inner root length 7 (6–7; *n* = 10); outer root length 4 (*n* = 10); point length 1 (*n* = 10). Dorsal bar 33 (33–35; *n* = 10) wide. Ventral bar 17 (15–18; *n* = 10) wide, 7 (6–8; *n* = 5) long, three-armed, poorly sclerotized or not observed. Hooks with delicate point, depressed thumb, shank comprised of 2 subunits (proximal subunit expanded); hook lengths: pair I 16 (15–16; *n* = 3), pair II 19 (19–20; *n* = 3), pair III 23 (22–24; *n* = 3), pair IV 23 (22–25; *n* = 3), pair V 15 (14–16; *n* = 3), pair VI 20 (19–20; *n* = 3), pair VII 20 (19–21; *n* = 3). FH loop about 0.75 times the distal shank length. MCO composed of basally articulated copulatory tube and accessory piece; total length 28 (27–29; *n* = 10). Copulatory tube 31 (30–32; *n* = 10) long; base with slightly frilled flange; shaft arcing, tapering terminally. Accessory piece subterminally divided and merged to form triangle serving as a guide for distal portion of copulatory tube. Vagina a tube with curled distal end and proximal cap.

*Remarks*: *Dactylogyrus leptus* n. sp. could be confused with *Dactylogyrus vranoviensis* Ergens, 1956, a gill parasite of *Squalius cephalus* (Linnaeus) (Danube, Oder and Elbe Rivers) [58], by having similar anchors and a similar dorsal bar. Anchors with a relatively short inner root, wide base, markedly elongate shaft with angular bend at its proximal quarter, and reduced point are common to both species. The ventral bar in the new species, although size-reduced as in *D. vranoviensis*, morphologically slightly resembles that of *Dactylogyrus dirigerus* Gussev, 1966 and *Dactylogyrus elegantis* Gussev, 1966, both parasites of *Chondrostoma* spp. in Central and Eastern Europe [4, 31, 58]. Unlike other *Dactylogyrus* species possessing a three-armed ventral bar, all three arms in the three species are almost the same size and are characterized by pointed ends (especially the transverse arms). The MCO of *D. leptus* n. sp. is intermediate between the “nanus” and “chondrostomi” types (i.e. the distal triangular widening of the accessory piece is elongated backwards along the tube to its initial part; it is short and claw-shaped [58]). It is most similar to that of *Dactylogyrus folkmanovae* Ergens, 1956 [58] by having an accessory piece with subterminal bifurcation but differs from it by having a more robust sheath-like terminal backwards elongation (*vs.* thin, hook-shaped backwards elongation in *D. folkmanovae*).

### Genetic divergences of *Dactylogyrus* species

In order to clearly document the specimens from which DNA sequences were obtained in this study, morphological vouchers (hologenophores) of the newly described *Dactylogyrus* species were deposited in publicly accessible collections (IPCAS, MNHN). Illustrations of the MCOs directly taken from the hologenophores are presented in Figure 13. Computations of genetic distances were performed for each genetic segment individually (partial gene coding 18S rRNA, partial gene coding 28S rRNA, and the ITS1 region). Alignments of 446, 770, and 390 positions, respectively, were used for the analyses. All positions containing gaps or missing data were eliminated from the alignments. The morphologically almost indistinguishable species *D. romuli* n. sp. and *D. remi* n. sp. exhibited moderate genetic divergence (*p-*distance = 0.2% in 18S, 2.3% in 28S, and 5.1% in ITS1). Genetic divergence was also investigated between phylogenetically and morphologically similar *Dactylogyrus* spp. parasitizing *Pachychilon* spp. (namely *D. recisus* n. sp., *D. martinovici* and *D. petkovici*). *Dactylogyrus recisus* n. sp. was genetically distant from *D. martinovici* and *D. petkovici* by 0.2% in partial 18S, by 1.4% and 1.7% in partial 28S, respectively and by 5.6% and 7.9% in ITS1, respectively. All pair-wise genetic distances are shown in Supplementary Tables 1–3.

### Phylogenetic position of the newly described *Dactylogyrus* species

**Figure 14:**
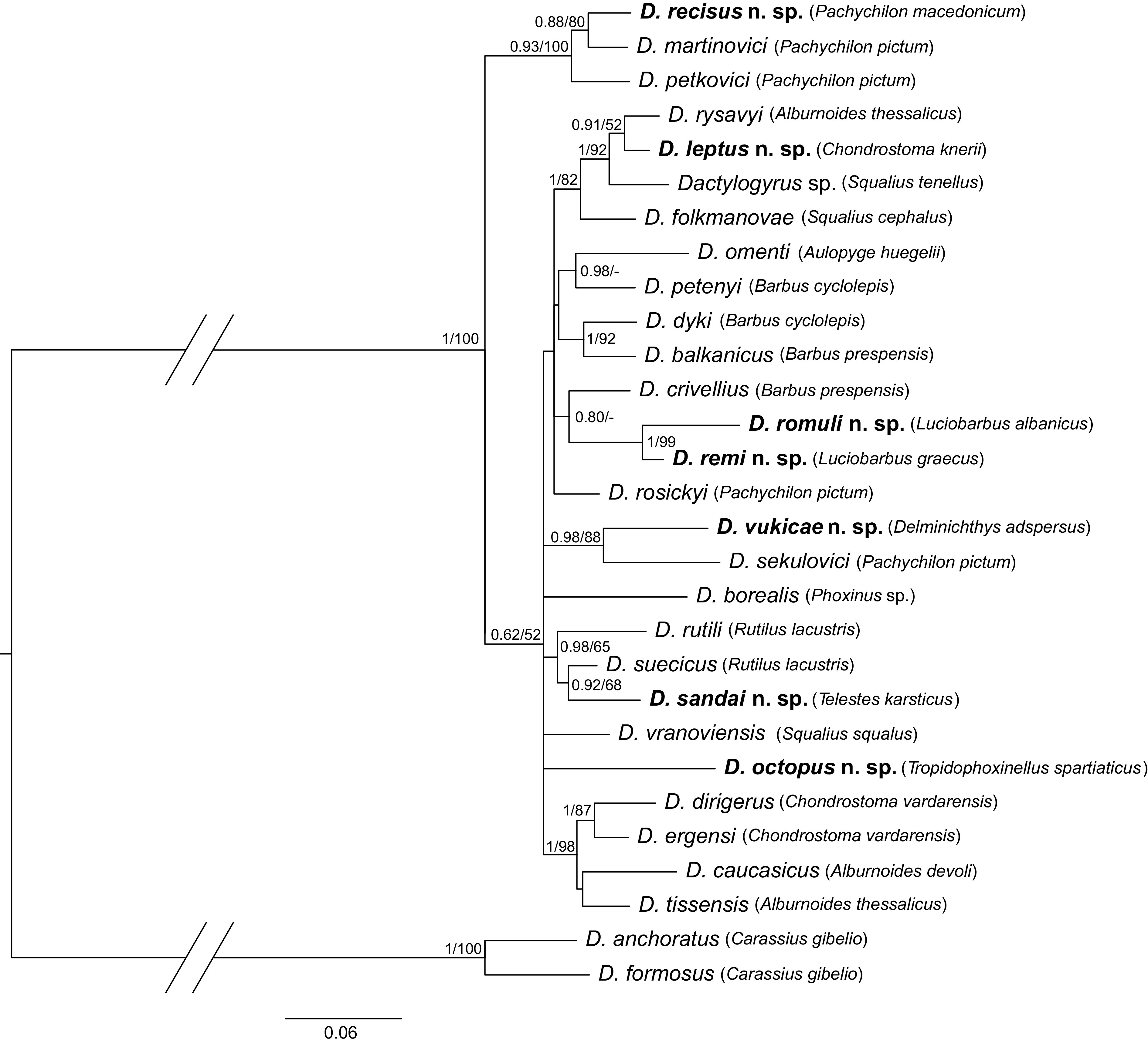
Phylogenetic tree of 29 *Dactylogyrus* species (hosts in brackets) constructed by Bayesian Inference (BI). The tree is based on the combined DNA sequences of partial genes coding 18S rRNA, 5.8S rDNA, 28S rRNA and the entire ITS1 region. Values along branches indicate posterior probabilities and bootstrap values for each node resulting from Bayesian Inference and Maximum likelihood (ML) analyses, respectively. Values <0.80 for BI and <50% for ML are indicated by dashes.

The final concatenated alignment of DNA sequences of 29 species contained 1647 unambiguous nucleotide positions. The optimal evolutionary models were K80 + I for partial 18S rDNA (446 bp), SYM + G for the ITS1 region (410 bp) and TIM2 + I + G for partial 28S rDNA (791 bp). Both phylogenetic analyses (ML and BI – Fig. 14) provided trees with congruent topologies and differed only in their node support values. Several well-to-moderately supported groups were revealed in the lineage comprising *Dactylogyrus* parasitizing cyprinoids. The first well-supported group was formed by two *Dactylogyrus* species from *Pachychilon pictum* (namely *D. martinovici* and *D. petkovici*) and *D. recisus* n. sp. collected from *P. macedonicum*. *Dactylogyrus leptus* n. sp. formed a well-supported group with *Dactylogyrus rysavyi* Ergens, 1970 from *Alburnoides thessalicus* Stephanidis and an undescribed *Dactylogyrus* sp. from Balkan *S. tenellus*. *Dactylogyrus folkmanovae*, a species parasitizing many *Squalius* spp. in Europe, was in basal position in this clade. Two new *Dactylogyrus* species from Balkan *Luciobarbus* spp. clustered together with *D. crivellius* Dupont & Lambert, 1987, a species strictly parasitizing *Barbus* spp. throughout Europe; however, the clade was only moderately supported using BI. All three species share similar haptoral sclerites (the same type of ventral bar). *Dactylogyrus vukicae* n. sp. was phylogenetically proximal to *Dactylogyrus sekulovici* Ergens, 1970 from *P. pictum*. *Dactylogyrus sandai* n. sp. grouped with *D. suecicus* and *D. rutili* (both common species of *Rutilus* spp.), which share similar morphological features with the newly described species. *Dactylogyrus octopus* n. sp. grouped with the other *Dactylogyrus* spp. from leuciscids; however, its position within the clade was not resolved.

## Discussion

Recent studies on Mediterranean cypriniforms suggest that the species diversity and endemicity of these fishes is higher than previously estimated [6, 7, 12, 16]. Considering the high host specificity of *Dactylogyrus* spp., the discovery of new species parasitizing Mediterranean cypriniforms can likewise be expected. A recent study carried out in the Balkan Peninsula [4] revealed eight potentially new *Dactylogyrus* species on two species of cyprinids (*L. albanicus*, and *L. graecus*) and five species of leuciscids (*C. knerii*, *D. adspersus*, *P. macedonicum*, *T. spartiaticus*, and *T. karsticus*). In the present paper, seven of these species are described on the basis of integrated morphological and molecular data; the *Dactylogyrus* species found on *S. tenellus* was not identified to species level due to an insufficient number of specimens.

Two pseudocryptic *Dactylogyrus* species, *D. romuli* n. sp. and *D. remi* n. sp., are described from *L. albanicus* and *L. graecus*, respectively, the only two species of *Luciobarbus* that are native in the Balkans. The highly diversified *Luciobarbus* comprises more than 30 species widely distributed along rivers draining into the Persian Gulf and the Mediterranean, Caspian, and Black seas [40]. To date, a total of 51 *Dactylogyrus* species are known from 29 species of *Luciobarbus* [47, 58, 59]. Most records come from Morocco and Spain, the regions with the highest diversity in *Luciobarbus* species [15, 43]. Except for three species (*D. balistae* Vicente, 1981, *D. guirensis* El Gharbi, Birgi & Lambert, 1994, and *D. legionensis* Gonzalez-Lanza & Alvarez-Pellitero, 1982), in which the ventral bar is absent, *Dactylogyrus* species infecting hosts of *Luciobarbus* are, on the basis of the shape of the ventral bar, grouped into four morphological types: with a rod-shaped, omega-shaped, inverted T-shaped, or cross-shaped ventral bar [58]. Both new *Dactylogyrus* spp. from Balkan *Luciobarbus* spp. belong to the group characterized by a cross-shaped ventral bar, where the anterior arm is bifurcated and the posterior arm is split or terminally frayed (= five radial type). *Dactylogyrus* spp. with this type of ventral bar have been recorded on *Luciobarbus* spp. inhabiting Morocco and the region around the Caspian Sea (Iran, Kazakhstan, East Transcaucasus) [58], and on *Aulopyge huegelii* Heckel in the Balkans [3]. Interestingly, the “Moroccan” group and the “Caspian” group of *Dactylogyrus* spp. with a five radial ventral bar are characterized by different types of MCO, i.e. the “chondrostomi” and “kulwieci” types, respectively [58]. The MCOs of the new *Dactylogyrus* spp. from Balkan *Luciobarbus* spp. could be classified as the “kulwieci” type (see Remarks for *D. romuli* n. sp.), which suggests that these new species are more closely related to the “Caspian” group of *Dactylogyrus* spp. than to the “Moroccan” one. *Dactylogyrus romuli* n. sp. and *D. remi* n. sp. are morphologically nearly indistinguishable. It is, therefore, unsurprising that our phylogenetic analyses showed these two species as closely related siblings (twin species), suggesting their recent common ancestry. Populations of *L. albanicus* (host of *D. romuli* n. sp.) and *L. graecus* (host of *D. remi* n. sp.) have recently lived in allopatry, although very close to each other in some regions of Central Greece [20], and represent different evolutionary lineages. According to Yang et al. [89], *L. albanicus* is phylogenetically closer to Middle Eastern and North African species of *Luciobarbus*, and *L. graecus* is closely related to *L. lydianus* from Turkey [87] and the Iberian lineage of *Luciobarbus* spp. The close relationship of *D. romuli* n. sp. and *D. remi* n. sp. and their occurrence on *L. albanicus* and *L. graecus*, respectively, may reflect historical contact between these two host species associated with parasite duplication (or intrahost speciation linked to reproductive isolation) followed by host switching. However, a more intensive survey of *Dactylogyrus* spp. parasitizing Balkan *Luciobarbus* spp. is needed to justify this assumption.

*Dactylogyrus recisus* n. sp. is described from the Greek *P. macedonicum*. Currently, only two species are recognized in this evolutionarily old leuciscid genus [52, 90], namely *P. macedonicum* (Gallikos to Pinios drainages [40]) and *P. pictum* (Drin to Aoos drainages [40]). Up to now, *Dactylogyrus* species have been reported only from *P. pictum* (i.e. *D. ivanovici, D. martinovici*, *D. petkovici*, *D. rosickyi*, and *D. sekulovici*) [23]. The relatively large genetic distances among some of these five *Dactylogyrus* species, as well as phylogenetic reconstructions ([4]; present study), suggest that *Dactylogyrus* spp. parasitizing *P. pictum* belong to four distinct lineages within *Dactylogyrus*, and thus have different origins probably resulting from multiple host switching. *Dactylogyrus recisus* n. sp. from *P. macedonicum* is phylogenetically closely related to *D. martinovici* and *D. petkovici* (Fig. 14). The haptoral structures of these three *Dactylogyrus* species share a similar morphology (compare Figure 8 with Figures 163 and 164 in Pugachev et al. [58]). This ties in with the suggestion of Šimková et al. [76] that *Dactylogyrus* spp. parasitizing phylogenetically closely related cyprinid species possess similar adaptations in the morphology of the haptor. On the other hand, there is no similarity among these three species with respect to the morphology of their MCOs. It has been hypothesized that monogenean species occupying the same microhabitats are reproductively isolated by the morphological differentiation of their MCOs, which probably avoids hybridization among the respective species [36, 64, 65, 73]. Although *P. macedonicum* and *P. pictum* inhabit different river drainages (see also fish collection localities in Benovics et al. [4]), it is possible that *D. recisus* n. sp., *D. martinovici* and *D. petkovici* evolved from a common *Dactylogyrus* ancestor parasitizing species of *Pachychilon*, which diversified through parasite duplication (i.e., intrahost speciation) followed by reproductive isolation.

*Telestes karsticus,* an endemic species inhabiting streams in Croatia [46], was found to be a host of *D. sandai* n. sp. *Telestes* currently comprises 14 species [8], and, of these, eight are found in Croatia [46]. Up to now, only one species of *Dactylogyrus*, *D. soufii* Lambert, 1977, has been reported on *Telestes souffia* (syn. *T. agassizii*) [58], a species occurring in the Danube and Rhone drainages [17]. The reconstruction of *Dactylogyrus* phylogeny focussing on species parasitizing cyprinoids in the Balkans showed that *D. sandai* n. sp. grouped with *D. suecicus* and *D. rutili* (both common species on *Rutilus* spp.). All three species share similar morphology regarding the haptoral structures and the same basic structure of the MCO. The phylogenetic proximity of *D. sandai* n. sp. and *D. suecicus* and the morphological similarities in MCOs between *D. sandai* n. sp. and the above mentioned *Dactylogyrus* spp. from Balkan *Rutilus* spp. suggests that *D. sandai* n. sp. originated from a recent host switch from widely distributed species of *Rutilus*.

*Tropidophoxinellus* is a small genus of two Greek (*T. hellenicus*, *T. spartiaticus*) and two North African (*T. callensis*, *T. chaignoni*; both formerly assigned to *Pseudophoxinus*) species of leuciscids [25, 52] that had not previously been investigated for monogeneans. On the gills of *T. spartiaticus*, we described *D. octopus* n. sp., whose phylogenetic position among other *Dactylogyrus* spp. parasitizing leuciscids was not resolved in our analysis. According to morphological [5, 90] and molecular data [52], species of *Tropidophoxinellus* are phylogenetically close to those of *Scardinius*. Interestingly, three *Dactylogyrus* species (*D. difformis* Wagener, 1857, *D. difformoides* Gläser & Gussev, 1967, and *D. izjumovae* Gussev, 1966) recorded on *Scardinius erythrophthalmus* (Linnaeus) show some similarities in haptoral morphology to *D. octopus* n. sp.; more specifically, all four species share the same type of dorsal anchor-bar complex and inverted T-shaped ventral bar with transverse arms resembling curly brackets. Benovics et al. [4] proposed that the above-mentioned *Dactylogyrus* spp. from *S. erythrophthalmus* form a highly-supported monophyletic group but are genetically distant from *D. octopus* n. sp. This suggests, in accordance with Šimková et al. [76], that the similarity in haptoral morphology between *D. octopus* n. sp. and *Dactylogyrus* spp. from *S. erythrophthalmus* is a result of adaptation to similar environments provided by phylogenetically closely related hosts, i.e. *Tropidophoxinellus* and *Scardinius*.

*Dactylogyrus vukicae* n. sp. is described from *D. adspersus*. A new species was expected to be found as no monogeneans had yet been described from this host genus. *Delminichthys* is one of the geographically most isolated genera of leuciscids. Species of this genus inhabit small karstic streams of the central Adriatic region [24]. According to Perea et al. [52], the independent evolution of *Delminichthys* began 14 MYA, when it separated from *Pelasgus*, a genus whose representatives were previously assigned to the southern Balkan *Pseudophoxinus* spp. [24, 40]. Due to a long period of separate evolutionary history [52], it is expected that species of *Delminichthys* host their own unique fauna of monogeneans – i.e., fauna that does not occur on other genera within the Leuciscidae. Indeed, *D. vukicae* n. sp. possesses a morphologically unique ventral bar that cannot be assigned to any of the 16 morphological types proposed for *Dactylogyrus* spp. so far reported from Palaearctic fishes (see [58]). The molecular data provided in this paper shows that *D. vukicae* n. sp. is phylogenetically close to *D. sekulovici* from *P. pictum*. A posteriori comparison of the hologenophores of these two *Dactylogyrus* spp. revealed no obvious morphological resemblance between their haptoral structures. However, both species share similarity in the morphology of their MCOs – specifically, the thin-walled base of the copulatory tube is bulbous with a medial finger-like thickening. According to Perea et al. [52], *D. adspersus* and *P. pictum* are representatives of phylogenetically unrelated ancient lineages, but have a similar geographical distribution. The phylogenetic proximity of *D. vukicae* n. sp. and *D. sekulovici* suggests the host-switching of parasites between these two leuciscid hosts living in sympatry in the central Adriatic region.

*Dactylogyrus leptus* n. sp. is described from *C. knerii*, a species endemic to the Neretva River (Bosnia and Herzegovina, Croatia) [29]. Species of *Chondrostoma* are distributed from the Rhine, Danube, and Po basins to the east, reaching southwest Iran [63]. *Chondrostoma knerii* belongs to the Italo-Balkanic group together with the Italian *Chondrostoma soetta* Bonaparte and the Bosnian *Chondrostoma phoxinus* Heckel [63]. To date, 21 *Dactylogyrus* species have been recorded on the gills of ten species of *Chondrostoma* [4, 47, 58]. The present phylogenetic analysis showed that *D. leptus* n. sp. forms a well-supported group with *D. rysavyi* from *A. thessalicus* and undescribed *Dactylogyrus* sp. from *S. tenellus*, an endemic species in the Balkans. *Dactylogyrus folkmanovae* Ergens, 1956, parasitizing many *Squalius* spp. in Europe, appears in a sister position to this clade. The morphological similarities in MCOs between *D. leptus* n. sp. and the above-mentioned *Dactylogyrus* spp. from *Squalius* spp. (see also remarks to *D. leptus* n. sp.) suggest that *D. leptus* n. sp. originated from a host-switch from widely distributed species of *Squalius*, and then adapted its haptoral structures to its new host species.

## Conclusion

*Dactylogyrus* is the most speciose genus (>900 nominal species; [28]) among helminth parasites and it is clear that the number of recorded *Dactylogyrus* species will continue to increase [13]. Since *Dactylogyrus* species exhibit remarkably high host specificity, the extraordinary species richness of this super-genus seems to be predictable from the high diversity of cypriniform hosts. If we leave out the number of regions/hosts unexplored for these parasites, a significant proportion of *Dactylogyrus* diversity may be hidden behind unrecognized cryptic species. The present study illustrates that some species considered as cryptic might be designated as pseudocryptic after a posteriori detailed morphological examination, as happened with *D. romuli* n. sp. and *D. remi* n. sp. In many groups, the morphological differentiation of closely related species may be difficult even for specialists, because a long time lag may exist between the primary genetic speciation and morphological differentiation [37]. Thus, for accurate species characterization, it is particularly important to acquire both morphological and molecular data from the same individual specimens, ideally along with illustrations of taxonomically important structures directly taken from hologenophores.

## Supplementary Material

Supplementary material is available at https://www.parasite-journal.org/10.1051/parasite/2020059/olm *Supplementary Table 1*. Pair-wise genetic distances for 28S sequences of *Dactylogyrus* species.
*Supplementary Table 2*. Pair-wise genetic distances for 18S sequences of *Dactylogyrus* species.
*Supplementary Table 3*. Pair-wise genetic distances for ITS1 sequences of *Dactylogyrus* species.
