# Supplementary material for: Uncovering the diversity of monogeneans (Platyhelminthes) on endemic cypriniform fishes of the Balkan Peninsula: new species of Dactylogyrus and comments on their phylogeny and host-parasite associations in a biogeographic context
Source: Parasite. 2020 Nov 24;27:66. doi: 10.1051/parasite/2020059 (PMC7685236; doi:10.1051/parasite/2020059)
Supplement: Supplementary file 1 — Supplementary Table 1. Pair-wise genetic distances for 28S sequences of Dactylogyrus species. Supplementary Table 2. Pair-wise genetic distances for 18S sequences of Dactylogyrus species. Supplementary Table 3. Pair-wise genetic distances for ITS1 sequences of Dactylogyrus species. [file parasite-27-66-s1.pdf]

Uncovering diversity of monogeneans (Platyhelminthes) on endemic cypriniform fishes of the Balkan Peninsula, with descriptions of new species and comments on their phylogeny and host-parasite associations in biogeographic context

Eva Řehulková, Michal Benovics, Andrea Šimková

PARASITE – supplementary data

Supplementary table 1. Pair-wise genetic distances for 28S sequences *of Dactylogyrus* species

	***Dactylogyrus* spp.**	**1**	**2**	**3**	**4**	**5**	**6**	**7**	**8**	**9**	**10**	**11**	**12**	**13**	**14**	**15**	**16**	**17**	**18**	**19**	**20**	**21**	**22**	**23**	**24**	**25**	**26**
**1**	*D. balkanicus*																										
**2**	*D. borealis*	0,034																									
**3**	*D. caucasicus*	0,023	0,031																								
**4**	*D. crivellius*	0,021	0,038	0,029																							
**5**	*D. dirigerus*	0,023	0,032	0,016	0,021																						
**6**	*D. dyki*	0,014	0,035	0,022	0,019	0,022																					
**7**	*D. ergensi*	0,023	0,029	0,018	0,026	0,016	0,019																				
**8**	*D. folkmanovae*	0,018	0,026	0,018	0,021	0,021	0,019	0,018																			
**9**	*D. leptus* n. sp.	0,026	0,036	0,029	0,029	0,029	0,025	0,023	0,018																		
**10**	*D. martinovici*	0,040	0,036	0,040	0,048	0,043	0,043	0,045	0,038	0,047																	
**11**	*D. octopus* n. sp.	0,042	0,045	0,042	0,047	0,044	0,043	0,042	0,042	0,044	0,048																
**12**	*D. omenti*	0,029	0,034	0,026	0,031	0,031	0,030	0,029	0,021	0,030	0,039	0,035															
**13**	*D. petenyi*	0,026	0,031	0,029	0,026	0,026	0,025	0,021	0,016	0,022	0,044	0,042	0,022														
**14**	*D. petkovici*	0,042	0,029	0,039	0,045	0,042	0,042	0,036	0,034	0,043	0,023	0,049	0,035	0,038													
**15**	*D. recisus* n. sp.	0,044	0,032	0,039	0,048	0,042	0,044	0,042	0,036	0,048	0,014	0,049	0,040	0,043	0,017												
**16**	*D. remi* n. sp.	0,031	0,035	0,029	0,029	0,026	0,030	0,034	0,029	0,039	0,047	0,039	0,034	0,034	0,045	0,048											
**17**	*D. romuli* n. sp.	0,043	0,040	0,043	0,040	0,039	0,042	0,043	0,038	0,048	0,061	0,055	0,045	0,043	0,056	0,062	0,023										
**18**	*D. rosickyi*	0,018	0,036	0,026	0,016	0,021	0,019	0,023	0,016	0,021	0,044	0,044	0,029	0,021	0,043	0,045	0,029	0,040									
**19**	*D. rutili*	0,026	0,032	0,022	0,030	0,022	0,026	0,022	0,022	0,025	0,036	0,036	0,030	0,027	0,036	0,034	0,032	0,044	0,027								
**20**	*D. rysavyi*	0,023	0,034	0,026	0,026	0,029	0,025	0,029	0,016	0,010	0,044	0,042	0,025	0,022	0,040	0,045	0,031	0,043	0,018	0,025							
**21**	*D. sandai* n. sp.	0,029	0,038	0,030	0,031	0,029	0,022	0,027	0,023	0,023	0,044	0,043	0,032	0,029	0,040	0,045	0,035	0,040	0,029	0,029	0,022						
**22**	*D. sekulovici*	0,042	0,044	0,042	0,044	0,039	0,045	0,042	0,036	0,038	0,044	0,052	0,043	0,036	0,044	0,045	0,051	0,061	0,036	0,034	0,035	0,048					
**23**	*D. suecicus*	0,019	0,032	0,025	0,025	0,023	0,021	0,022	0,017	0,025	0,040	0,040	0,027	0,025	0,036	0,042	0,032	0,036	0,025	0,018	0,025	0,019	0,040				
**24**	*D. tissensis*	0,031	0,039	0,018	0,034	0,021	0,027	0,016	0,023	0,034	0,047	0,049	0,034	0,031	0,043	0,045	0,039	0,051	0,029	0,030	0,031	0,031	0,044	0,027			
**25**	*D. vranoviensis*	0,025	0,036	0,027	0,025	0,027	0,025	0,025	0,012	0,018	0,043	0,045	0,027	0,019	0,040	0,042	0,034	0,043	0,014	0,029	0,016	0,029	0,031	0,025	0,029		
**26**	*D. vukicae* n. sp.	0,047	0,048	0,045	0,052	0,047	0,051	0,048	0,038	0,035	0,053	0,058	0,045	0,047	0,055	0,051	0,058	0,066	0,043	0,034	0,032	0,045	0,032	0,043	0,051	0,035	
**27**	*Dactylogyrus* sp.	0,026	0,036	0,029	0,026	0,026	0,022	0,023	0,016	0,012	0,042	0,040	0,026	0,018	0,038	0,043	0,029	0,038	0,018	0,025	0,012	0,018	0,036	0,022	0,034	0,017	0,039

Supplementary table 2. Pair-wise genetic distances for 18S sequences of *Dactylogyrus* species

	***Dactylogyrus* spp.**	**1**	**2**	**3**	**4**	**5**	**6**	**7**	**8**	**9**	**10**	**11**	**12**	**13**	**14**	**15**	**16**	**17**	**18**	**19**	**20**	**21**	**22**	**23**	**24**	**25**	**26**
**1**	*D. balkanicus*																										
**2**	*D. borealis*	0,027																									
**3**	*D. caucasicus*	0,022	0,018																								
**4**	*D. crivellius*	0,020	0,016	0,011																							
**5**	*D. dirigerus*	0,025	0,016	0,011	0,007																						
**6**	*D. dyki*	0,013	0,020	0,016	0,009	0,013																					
**7**	*D. ergensi*	0,025	0,011	0,007	0,009	0,009	0,013																				
**8**	*D. folkmanovae*	0,020	0,011	0,007	0,004	0,004	0,009	0,004																			
**9**	*D. leptus* n. sp.	0,022	0,013	0,009	0,007	0,002	0,011	0,007	0,002																		
**10**	*D. martinovici*	0,020	0,011	0,007	0,004	0,004	0,009	0,004	0,000	0,002																	
**11**	*D. octopus* n. sp.	0,025	0,016	0,016	0,013	0,009	0,018	0,013	0,009	0,007	0,009																
**12**	*D. omenti*	0,025	0,020	0,016	0,013	0,009	0,013	0,013	0,009	0,007	0,009	0,013															
**13**	*D. petenyi*	0,020	0,016	0,011	0,009	0,009	0,009	0,009	0,004	0,007	0,004	0,013	0,004														
**14**	*D. petkovici*	0,020	0,011	0,007	0,004	0,004	0,009	0,004	0,000	0,002	0,000	0,009	0,009	0,004													
**15**	*D. recisus* n. sp.	0,018	0,013	0,009	0,007	0,007	0,011	0,007	0,002	0,004	0,002	0,011	0,011	0,007	0,002												
**16**	*D. remi* n. sp.	0,020	0,007	0,011	0,009	0,009	0,013	0,004	0,004	0,007	0,004	0,009	0,013	0,009	0,004	0,007											
**17**	*D. romuli* n. sp.	0,022	0,004	0,013	0,011	0,011	0,016	0,007	0,007	0,009	0,007	0,011	0,016	0,011	0,007	0,009	0,002										
**18**	*D. rosickyi*	0,022	0,013	0,009	0,007	0,002	0,011	0,007	0,002	0,000	0,002	0,007	0,007	0,007	0,002	0,004	0,007	0,009									
**19**	*D. rutili*	0,020	0,011	0,011	0,009	0,009	0,013	0,009	0,004	0,007	0,004	0,009	0,013	0,009	0,004	0,007	0,004	0,007	0,007								
**20**	*D. rysavyi*	0,020	0,011	0,007	0,004	0,004	0,009	0,004	0,000	0,002	0,000	0,009	0,009	0,004	0,000	0,002	0,004	0,007	0,002	0,004							
**21**	*D. sandai* n. sp.	0,025	0,016	0,011	0,009	0,009	0,013	0,009	0,004	0,007	0,004	0,013	0,013	0,009	0,004	0,007	0,009	0,011	0,007	0,004	0,004						
**22**	*D. sekulovici*	0,022	0,018	0,013	0,009	0,002	0,011	0,011	0,007	0,004	0,007	0,011	0,007	0,007	0,007	0,009	0,011	0,013	0,004	0,011	0,007	0,011					
**23**	*D. suecicus*	0,022	0,009	0,013	0,011	0,011	0,016	0,011	0,007	0,009	0,007	0,011	0,016	0,011	0,007	0,009	0,007	0,004	0,009	0,002	0,007	0,007	0,013				
**24**	*D. tissensis*	0,020	0,011	0,007	0,004	0,004	0,009	0,004	0,000	0,002	0,000	0,009	0,009	0,004	0,000	0,002	0,004	0,007	0,002	0,004	0,000	0,004	0,007	0,007			
**25**	*D. vranoviensis*	0,025	0,011	0,011	0,009	0,009	0,013	0,004	0,004	0,007	0,004	0,013	0,013	0,009	0,004	0,007	0,004	0,007	0,007	0,009	0,004	0,009	0,011	0,011	0,004		
**26**	*D. vukicae* n. sp.	0,025	0,025	0,020	0,018	0,018	0,013	0,018	0,013	0,016	0,013	0,020	0,018	0,013	0,013	0,016	0,018	0,020	0,016	0,018	0,013	0,018	0,016	0,020	0,013	0,018	
**27**	*Dactylogyrus* sp.	0,022	0,013	0,009	0,007	0,002	0,011	0,007	0,002	0,000	0,002	0,007	0,007	0,007	0,002	0,004	0,007	0,009	0,000	0,007	0,002	0,007	0,004	0,009	0,002	0,007	0,016

Supplementary table 3. Pair-wise genetic distances for ITS1 sequences of *Dactylogyrus* species

	***Dactylogyrus* spp.**	**1**	**2**	**3**	**4**	**5**	**6**	**7**	**8**	**9**	**10**	**11**	**12**	**13**	**14**	**15**	**16**	**17**	**18**	**19**	**20**	**21**	**22**	**23**	**24**	**25**	**26**
**1**	*D. balkanicus*																										
**2**	*D. borealis*	0,115																									
**3**	*D. caucasicus*	0,113	0,118																								
**4**	*D. crivellius*	0,090	0,149	0,126																							
**5**	*D. dirigerus*	0,115	0,141	0,100	0,105																						
**6**	*D. dyki*	0,064	0,110	0,103	0,082	0,121																					
**7**	*D. ergensi*	0,110	0,123	0,072	0,108	0,067	0,095																				
**8**	*D. folkmanovae*	0,121	0,172	0,151	0,110	0,128	0,110	0,133																			
**9**	*D. leptus* n. sp.	0,100	0,144	0,113	0,097	0,115	0,082	0,110	0,079																		
**10**	*D. martinovici*	0,138	0,164	0,162	0,136	0,169	0,138	0,151	0,154	0,141																	
**11**	*D. octopus* n. sp.	0,131	0,159	0,156	0,133	0,156	0,121	0,138	0,138	0,121	0,169																
**12**	*D. omenti*	0,126	0,187	0,136	0,118	0,149	0,118	0,141	0,144	0,123	0,172	0,162															
**13**	*D. petenyi*	0,087	0,133	0,126	0,092	0,128	0,090	0,118	0,115	0,105	0,138	0,138	0,138														
**14**	*D. petkovici*	0,146	0,177	0,172	0,149	0,179	0,144	0,167	0,151	0,149	0,074	0,164	0,177	0,141													
**15**	*D. recisus* n. sp.	0,146	0,164	0,167	0,144	0,174	0,146	0,151	0,169	0,149	0,056	0,174	0,190	0,156	0,079												
**16**	*D. remi* n. sp.	0,108	0,138	0,141	0,082	0,131	0,103	0,123	0,118	0,113	0,144	0,133	0,144	0,105	0,146	0,156											
**17**	*D. romuli* n. sp.	0,123	0,151	0,154	0,105	0,136	0,113	0,131	0,131	0,136	0,164	0,144	0,162	0,126	0,162	0,179	0,051										
**18**	*D. rosickyi*	0,072	0,123	0,103	0,074	0,092	0,064	0,087	0,105	0,082	0,115	0,113	0,105	0,074	0,131	0,131	0,090	0,113									
**19**	*D. rutili*	0,103	0,141	0,154	0,131	0,126	0,118	0,133	0,141	0,123	0,164	0,128	0,172	0,133	0,174	0,156	0,121	0,146	0,090								
**20**	*D. rysavyi*	0,110	0,144	0,131	0,097	0,121	0,100	0,121	0,085	0,051	0,144	0,131	0,141	0,113	0,149	0,151	0,108	0,131	0,092	0,141							
**21**	*D. sandai* n. sp.	0,110	0,138	0,113	0,108	0,072	0,108	0,077	0,133	0,113	0,149	0,133	0,144	0,115	0,169	0,151	0,131	0,144	0,077	0,121	0,128						
**22**	*D. sekulovici*	0,121	0,146	0,131	0,115	0,131	0,105	0,123	0,131	0,115	0,146	0,144	0,154	0,110	0,159	0,151	0,136	0,133	0,103	0,151	0,128	0,118					
**23**	*D. suecicus*	0,085	0,126	0,100	0,087	0,079	0,090	0,079	0,113	0,097	0,133	0,113	0,121	0,103	0,141	0,141	0,103	0,123	0,056	0,087	0,115	0,062	0,103				
**24**	*D. tissensis*	0,095	0,133	0,082	0,100	0,077	0,097	0,064	0,126	0,105	0,133	0,121	0,138	0,100	0,154	0,138	0,115	0,131	0,074	0,128	0,113	0,085	0,105	0,074			
**25**	*D. vranoviensis*	0,087	0,108	0,103	0,090	0,115	0,095	0,095	0,128	0,113	0,146	0,113	0,141	0,103	0,141	0,149	0,097	0,115	0,085	0,113	0,108	0,092	0,131	0,082	0,095		
**26**	*D. vukicae* n. sp.	0,115	0,149	0,123	0,131	0,123	0,123	0,115	0,133	0,128	0,162	0,151	0,162	0,128	0,177	0,154	0,138	0,141	0,108	0,133	0,141	0,110	0,095	0,103	0,092	0,128	
**27**	*Dactylogyrus* sp.	0,110	0,146	0,138	0,100	0,128	0,100	0,131	0,092	0,067	0,149	0,123	0,144	0,115	0,159	0,159	0,103	0,126	0,100	0,133	0,079	0,133	0,133	0,113	0,121	0,118	0,146
